# The Tumor Immune Profile of Murine Ovarian Cancer Models: An Essential Tool for Ovarian Cancer Immunotherapy Research

**DOI:** 10.1158/2767-9764.CRC-22-0017

**Published:** 2022-06-09

**Authors:** Galaxia M. Rodriguez, Kristianne J.C. Galpin, David P. Cook, Edward Yakubovich, Vincent Maranda, Elizabeth A. Macdonald, Juliette Wilson-Sanchez, Anjali L. Thomas, Joanna E. Burdette, Barbara C. Vanderhyden

**Affiliations:** 1 Cancer Therapeutics Program, Ottawa Hospital Research Institute, Ottawa, Ontario, Canada.; 2 Department of Cellular and Molecular Medicine, University of Ottawa, Ottawa, Ontario, Canada.; 3 Department of Pharmaceutical Sciences, College of Pharmacy, University of Illinois at Chicago, Chicago, Illinois.

## Abstract

Epithelial ovarian cancer (EOC) is the most lethal gynecologic cancer with an imperative need for new treatments. Immunotherapy has had marked success in some cancer types; however, clinical trials studying the efficacy of immune checkpoint inhibitors for the treatment of EOC benefited less than 15% of patients. Given that EOC develops from multiple tissues in the reproductive system and metastasizes widely throughout the peritoneal cavity, responses to immunotherapy are likely hindered by heterogeneous tumor microenvironments (TME) containing a variety of immune profiles. To fully characterize and compare syngeneic model systems that may reflect this diversity, we determined the immunogenicity of six ovarian tumor models *in vivo*, the T and myeloid profile of orthotopic tumors and the immune composition and cytokine profile of ascites, by single-cell RNA sequencing, flow cytometry, and IHC. The selected models reflect the different cellular origins of EOC (ovarian and fallopian tube epithelium) and harbor mutations relevant to human disease, including *Tp53* mutation, PTEN suppression, and constitutive KRAS activation. ID8-p53^−/−^ and ID8-C3 tumors were most highly infiltrated by T cells, whereas STOSE and MOE-PTEN/KRAS tumors were primarily infiltrated by tumor-associated macrophages and were unique in MHC class I and II expression. MOE-PTEN/KRAS tumors were capable of forming T-cell clusters. This panel of well-defined murine EOC models reflects some of the heterogeneity found in human disease and can serve as a valuable resource for studies that aim to test immunotherapies, explore the mechanisms of immune response to therapy, and guide selection of treatments for patient populations.

**Significance:**

This study highlights the main differences in the immunogenicity and immune composition found in six different models of orthotopic ovarian cancer as an essential tool for future preclinical investigations of cancer immunotherapy.

## Introduction

Epithelial ovarian cancer (EOC) is the fifth most common cause of cancer-related deaths among North American women, and the most lethal gynecologic cancer (1). High-grade serous cancer (HGSC) makes up 70% of EOC with a 5-year survival of less than 45%. Standard treatment involves platinum-based chemotherapy and surgical debulking; however, many patients become resistant to first-line chemotherapy and effective second-line therapies are limited (2). There is a pressing need for new therapies to treat recurrent and resistant disease.

The ovarian cancer tumor microenvironment (TME) is often infiltrated by immune cells, whose presence correlates with increased patient survival (3); specifically, a higher ratio of CD8^+^ T cells to regulatory T cells (Tregs; ref. 4). Conversely, loss of MHC-I expression or other components of the antigen presentation machinery is associated with poor survival (5, 6). This complex network of antitumoral and immunosuppressive activities includes T cells, Tregs, dendritic cells (DC), myeloid-derived suppressor cells (MDSC), and tumor-associated macrophages (TAM; ref. 7). Immune checkpoint inhibitors (ICI) are promising strategies to improve prognosis in patients with cancer (8); however, they have shown limited success in ovarian cancer, with response rates of only 5%–20% (9, 10). Ovarian cancers possess low/intermediate levels of mutation burden and neoantigen load (11, 12) which may limit the effectiveness of immunotherapies. Current attention is therefore focused on improving immunotherapeutic strategies using novel and combination treatments.

Immunocompetent mouse models are critical to understand efficacy of new therapies and mechanisms of resistance. Response to immunotherapy in transplantable syngeneic models correlated with MHC-I expression, while the composition of tumor-infiltrating immune cells was based on intrinsic (MHC-I, mutation burden, transplant location) and extrinsic characteristics (e.g., strain; refs. 13–15). Therefore, profiling of syngeneic models will help researchers select models to perform immunotherapy studies, and may guide patient stratification to maximize efficacy of treatment.

Roby and colleagues generated the first syngeneic model of ovarian cancer, ID8 cells (16), derived from mouse ovarian surface epithelial (OSE) cells have been successfully used for preclinical testing of immunotherapies (8). Recently, ID8 cells were edited to knock out genes commonly deleted in ovarian cancer, including *Trp53*, *Brca1*, and/or *Brca2,* increasing the preclinical relevance of this model as 96% of HGSC cases have *Trp53* mutations and 33% are *Brca* altered (17–19). We generated a spontaneously transformed cell line, STOSE, derived from the OSE of FVB/N mice (20); like ID8 cells, this model lacks mutations/deletions in *Trp53* (20). While tumors resulting from STOSE and ID8 cells histologically resemble HGSC (16, 20), their origins from the OSE model only a fraction of HGSC. To model HGSC from the fallopian tube, we generated a mouse oviductal epithelial (MOE) cell line (21), which J. Burdette's lab subsequently rendered tumorigenic by modifying PI3K/RAS signaling, which is altered in 45% of HGSC cases (17). Modifications included knockdown of *PTEN* (MOE-PTEN^shRNA^) alone or in combination with a common gain-of-function mutation (Arg273His) in *Trp53* (MOE-PTEN^shRNA^p53^R273H^), or with an activating mutation in *Kras* (MOE-PTEN^shRNA^KRAS^G12V^ (22, 23).

The goal of this study was to profile the immune composition of orthotopic tumors, ascites, and spleens of advanced stage tumor-bearing mice. We compared the different immune subsets in these tissues, expression of immunomodulatory markers, and immunogenicity of syngeneic models originating from both OSE and MOE and bearing clinically relevant mutations. This collection of models serves as a valuable resource to guide preclinical research by facilitating the selection of appropriate models to understand the mechanisms underlying response to immunotherapies and to suggest patient populations that may benefit from these treatments.

## Materials and Methods

### Study Design

The goal of this study was to characterize the TME of syngeneic mouse models of ovarian cancer. Mice were euthanized 4–5 days prior to humane endpoint for each model and tumor-naïve age-matched control mice were included to normalize between experiments. The immune composition was analyzed by flow cytometry using multiparameter settings. Image quantification was performed on IHC images identified by three-number codes and quantification macros were applied in an unbiased fashion to all sections stained for IHC on the same day with the same antibody. Detailed methods and sample processing are described below.

### Cell Lines

ID8-WT cells were provided by Kathy Roby (16). ID8-*Trp53^−^^/−^* F3 (ID8-p53^−/−^), ID8-*Trp53^−^^/^^−^Brca1*^−/−^, and ID8-*Trp53^−^^/^^−^Brca2*^−/−^ generated by CRISPR-Cas9–mediated knockout and ID8-C3 (CRISPR control) were generously provided by Iain McNeish and maintained as previously described (18, 19). STOSE cells were generated in our lab and maintained in MOSE media as reported (20). MOE cell lines were generated as described previously (21). MOE-PTEN^shRNA^ (MOE-PTEN), MOE-PTEN^shRNA^p53^R273H^ (MOE-PTEN/p53), and MOE-PTEN^shRNA^KRAS^G12V^ (MOE-PTEN/KRAS) were generously provided by Joanna Burdette (University of Illinois, Chicago) and maintained as previously described (22, 23). Cells were passaged a minimum of two times after thawing and maximum of ten times prior to *in vivo* studies. As ID8-WT cells, ID8 derivatives and MOE cells were acquired directly from their source, while STOSE cells were generated by our lab, separate short tandem repeat validation was not performed. *Mycoplasma* testing was performed prior to animal experiments (PlasmoTest negative as of February 12, 2019 prior to injections beginning in February 14, 2019).

For IFNγ treatment, cell lines were seeded in 6-well tissue culture plates (Corning) and treated with 500 pg/mL murine IFNγ (PeproTech) for 24 hours. Cells were incubated at 37°C with 5% carbon dioxide. No antibiotics were used in any cultures. *In vivo* experiments were performed with the selected cell lines able to form a primary tumor of sufficient size for flow cytometry analysis. The *Brca* mutants were excluded from the *in vivo* study as they had already undergone characterization of immune cell infiltration (18).

### Mouse Models and *In Vivo* Studies

Animal experiments were carried out using protocols approved by the Animal Care Committee at the University of Ottawa and conforming to or exceeding the standards defined by the Canadian Council on Animal Care. FVB/N mice (for STOSE and MOE cell lines) were acquired from Charles River and C57BL/6 mice (for ID8 and derivatives) were purchased from Jackson Laboratories. Intrabursal injections of cell lines (0.15 × 10^6^ cells) were performed as previously described (20). Intraperitoneal injections of 5 × 10^6^ cells were delivered to a subset of the mice. As the mice developing intrabursal tumors neared humane endpoint (4–5 days before anticipated endpoint), primary tumors, abdominal metastases, spleens, and ascites were collected for flow cytometry, IHC (frozen and fixed), immunofluorescence, qPCR, and single-cell RNA-sequencing analysis.

#### Immunogenicity

Cells were released from adherent cultures using trypsin (0.05% trypsin, 0.53 mmol/L EDTA), washed two times with PBS, counted to assess viability, and 5 × 10^6^ cells/mouse were injected intraperitoneally. Tumors were allowed to establish over a period of two weeks. At that time, a second set of cells were released from adherent cultures and irradiated at 100 Gy (ID8) or 60 Gy (STOSE and MOE cell lines), washed two times, and then 5 × 10^6^ cells/mouse (or PBS as control) were injected intraperitoneally. Mice were monitored for survival until a humane endpoint.

### Flow Cytometry

When present, ascites fluid was collected from mice. In other cases, peritoneal wash (PW) was collected following an intraperitoneal injection of 5 mL PBS-EDTA 1 mmol/L for 5 minutes with abdominal massage. Ascites or PW were filtered through a 70 μm filter to generate a single-cell suspension of peritoneal cells. Single-cell suspensions of spleens were derived by mechanical disruption in PBS + 2% FBS. Tumors (maximum 1 g) were cut into tiny pieces using a razor blade in 2.5 mL of digestion solution following the manufacturer's protocol using the mouse Tumor Dissociation Kit (Miltenyi Biotec) and a gentleMACS Octo Dissociator with heaters (protocol tumor 37°C_m_TDK_1). Following the dissociation protocol, the suspension was filtered, and remaining tumor pieces were further disrupted using a syringe plunger on a 70 μm filter to generate a single-cell suspension. All samples underwent blood cell lysis with ACK Lysing Buffer (VWR). Cells were counted to assess viability (Trypan blue exclusion) prior to staining. Cells were stained for viability (BD Biosciences Fixable Viability Stain, 510) at 1:500 per 3 × 10^7^ cells for 15 minutes at room temperature. Fc-blocking antibody was incubated with samples at 3.33 or 6.67 μL for every 2 × 10^7^ cells from the spleen or the tumors/ascites, respectively, for 5 minutes prior to extracellular staining (Supplementary Table S1) carried out at room temperature for 20 minutes. Samples were fixed in 1% paraformaldehyde (PFA) and stored overnight at 4°C until samples were analyzed on a BD Celesta flow cytometer by using multi-parameter settings.

For the panel of “myeloid-like” cells (see Supplementary Table S1), samples were incubated for 45 minutes at room temperature with fixation/permeabilization working solution (eBioscience) and then washed twice with 1× Permeabilization Buffer (eBioscience) intracellular staining. Anti-CD206 intracellular antibody was diluted in the permeabilization buffer and added to the “myeloid-like” panel samples for 40 minutes in the dark at room temperature. Samples were then washed with 1× permeabilization buffer and then with PBS + 2% FBS. Finally, samples were resuspended in 1% PFA and stored overnight at 4°C until analyzed by flow cytometry the following day. Data was analyzed by FlowJo software (v10.7.2, TreeStar).

### Single-cell RNA Sequencing

#### Single-cell RNA Sequencing Sample Preparation

Single-cell suspensions were obtained as described above (see Flow Cytometry section). Dead cells were removed from the single-cell suspension using the microbead-based Dead Cell Removal Kit (Miltenyi Biotec) according to the manufacturer's protocol. Briefly, <10^7^ cells were centrifuged at 300 × *g* for 10 minutes and resuspended in 100 μL of Dead Cell Removal MicroBeads. After 15 minutes of incubation at room temperature, the sample was passed through MACS MS columns (Miltenyi Biotec) and washed, collecting the flow-through of unlabeled live cells. After removal, each sample had >80% viable cells.

#### Single-cell RNA Sequencing Library Preparation and Sequencing

Single-cell suspensions were processed using the 10X Genomics Single Cell 3′ RNA-seq kit (v3), loaded to target a yield of 10,000 cells per sample. Cell cDNA libraries were prepared according to the manufacturer's protocol and libraries were assessed using the Fragment Analyzer (Agilent). Libraries were sequenced using the high-output 75 cycle kit on the NextSeq500 (Illumina) achieving approximately 20,000–25,000 reads per cell. For the STOSE tumor sample, we detected a median of 10,321 UMI counts per cell and a median of 3,076 genes. For the ID8 sample, we detected a median of 7,216 UMI counts per cell and a median of 2,517 genes.

#### Processing of Raw Sequencing Reads

Raw sequencing reads were processed using CellRanger v3.0.2 and the mm10 build of the mouse genome. Default settings were used for both libraries.

#### Data Quality Control and Processing

Quality control was performed independently on each sample and all main processing steps were performed with *Seurat* v3.0.2 (24). UMI count matrices from CellRanger were loaded into R and cells with fewer than 200 detected genes were removed. Cells with a high percentage (>25%) of mitochondria-associated transcripts were also removed. The expression values were then normalized with standard library size scaling and log transformation. The top 3,000 variable genes were detected using the variance stabilization transformation (vst) selection method in Seurat. Expression values were scaled, and cell-cycle scores and the percentage of mitochondrial reads were regressed out. Principal component analysis was then performed on the data prior to integrating the two tumor samples using the R package *Harmony.* The integrated embedding was then used to cluster cells (FindNeighbors dims = 1:40, FindClusters resolution = 0.3) and to a UMAP embedding of the data was generated (dims = 1:40). Differential gene expression was conducted by using the “subset” function in Seurat to subset relevant populations of comparison followed by applying the “FindMarkers” function in Seurat using a MAST test comparing ID8 and STOSE tumor subpopulations against each other. Myeloid and lymphocyte populations were identified using an unsupervised reference-based method (25) while cancer cells (*Krt14, Krt19, Msln, Amhr2*), fibroblasts (*Col1a1, Col1a2*), and endothelial cells (*Pecam, Flt1*) were identified using the indicated markers. Raw sequencing files and processed single-cell RNA sequencing (scRNA-seq) data are available at the NCBI GEO accession GSE183368.

#### ID8/STOSE Cancer Cell Identity

Projective nonnegative matrix factorization “scPNMF” package (26) was applied to either the ID8 or STOSE cancer cell populations, and then filtered out nonunique amplitude matrix genes to generate a gene set comprising the identity of either ID8 or STOSE cancer cells. Gene sets were validated by scoring every cancer cell in either ID8 or STOSE variants to show enrichment respective to the gene set and the cancer cells used to generate it.

#### ID8/STOSE Macrophages

A previously published microarray dataset by Jablonski and colleagues (2015; ref. 26) that identified either M0-, M1-, or M2-polarized macrophages was leveraged to generate unique gene-sets for each macrophage subpopulation. Standard normalization and scaling methodology was applied to the raw matrix, followed by filtering out genes undetected by the microarray, and then a series of *t* tests corrected with FDR against the log_2_FC difference means was used to generate a gene set of 306 unique M0 genes, 459 unique M1 genes, and 478 M2 genes. The macrophage population was subset from either ID8 or STOSE sequenced tumors and scored to validate the specificity and accuracy of these gene sets by enrichment.

#### Data and Materials Availability

Raw sequencing files and processed scRNA-seq data are available at the NCBI GEO accession GSE183368. All other data are available in the main text or the Supplementary Data.

### IHC and Immunofluorescence

For IHC, 5 μm sections of formalin-fixed paraffin-embedded tissue were deparaffinized in xylene and an alcohol gradient, and pressurized antigen retrieval was performed in a citrate buffer (antigen unmasking solution pH 6.0, Dako). Endogenous peroxidase activity was blocked by 10-minute incubation in 3% hydrogen peroxide. Following PBS washes, sections were blocked for nonspecific staining with a protein block (Dako) for 1 hour at room temperature. Immunostaining was performed in antibody diluent (Dako) using antibodies and conditions listed in Supplementary Table S2. Species-appropriate horseradish peroxidase–conjugated secondary antibodies (Vector Laboratories) were then added for 1 hour at room temperature. Sections were developed with 0.2% diaminobenzidine (DAB)/0.001% hydrogen peroxide solution (DAB, Sigma, D8001) for 5 minutes followed by counterstaining with hematoxylin (room temperature for 1 minute). Sections were then rehydrated in an alcohol gradient and xylene and mounted with permount (Thermo Fisher Scientific). Images were acquired using the Zeiss AxioScan Z1 (20× objective). Quantification was performed using ImageProPremier (cells/mm^2^) and Orbit Image Analysis (percent positive pixels %).

IF experiments were done using 7 μm sections of tissue snap frozen in Tissue-Tek O.C.T. compound. Sections stored at −80°C were brought to room temperature and fixed using ice-cold methanol (MHC-I, ab15681), ice-cold acetone (MHC-I, ab15680), or 2% PFA (CK8+18/MHC-II) for 20 minutes. Tissue sections were washed two times with PBS and blocked in 10% goat serum in PBS (+ 0.01% Triton-100 for CK8+18/MHC-II) for 1 hour at room temperature. Primary antibodies (Supplementary Table S2), followed by secondary antibodies of the appropriate species (Invitrogen), were diluted in 10% goat serum in PBS. Slides were mounted with Immu-Mount (Thermo Fisher Scientific), cured overnight, and visualized on the AxioSkop 2 MOT (Zeiss) microscope (40× objective).

### Quantitative RT-PCR

Cells were released from adherent cultures using trypsin (0.05% trypsin, 0.53 mmol/L EDTA) and washed with PBS before lysis. RNA was extracted according to the manufacturer's instructions with the Illustra RNAspin Mini Kit (GE Healthcare). RNA was then quantified and quality assessed using a NanoDrop 3300 Spectrometer (Thermo Fisher) and cDNA was prepared with iScript Reverse Transcription Supermix (Bio-Rad), using 1 μg of RNA. Relative gene expression was determined by quantitative PCR (qPCR) SYBR Green Supermix (Bio-Rad) and the Applied Biosystems 7500 Fast Real-Time PCR system to quantify gene expression using primers (Invitrogen) specific to our genes of interest (Supplementary Table S3). Gene expression was calculated as fold increase over untreated cells, normalized to the housekeeping gene *Rplp0*.

### LEGENDplex Bead-Based Immunoassay

Plasma was acquired from blood collected by cardiac puncture into heparin-coated capillary tubes (Microvette CB 300 Lh, Sarstedt). Ascites fluid was collected from mice, centrifuged at 2,000 rpm for 15 minutes, and supernatant was frozen at −80°C until assay. Ascites supernatant and plasma samples were diluted 1:2 in assay buffer and assayed according to the manufacturer's protocol for the LEGENDplex Mouse Cytokine Release Syndrome Panel (13-plex) Multi-Analyte Flow Assay Kit (BioLegend). Samples were acquired in duplicate the same day of staining, on a BD LSR Fortessa flow cytometer and analyzed using LEGENDplex Quognit software (BioLegend).

### Statistical Analysis

All graphs were prepared in Prism 9.0 or using R (ggplot2, pheatmap). Statistical analyses were performed with Prism 9.0 (GraphPad Software Inc.). Pairwise comparisons were made using a two-tailed Student *t* test. Multiple comparisons were performed using one-way ANOVA followed by Tukey multiple comparisons test. Results were considered statistically significant at *P* < 0.05. Data are presented as the means ± SD or SEM, as indicated. The *P* values of comparison between survival plots were calculated by log-rank (Mantel–Cox) tests using Kaplan–Meier plots.

## Results

### OSE-Derived STOSE Tumors Express MHC-I and are Preferentially Infiltrated by M2 Tumor-Associated Macrophages

**FIGURE 1:**
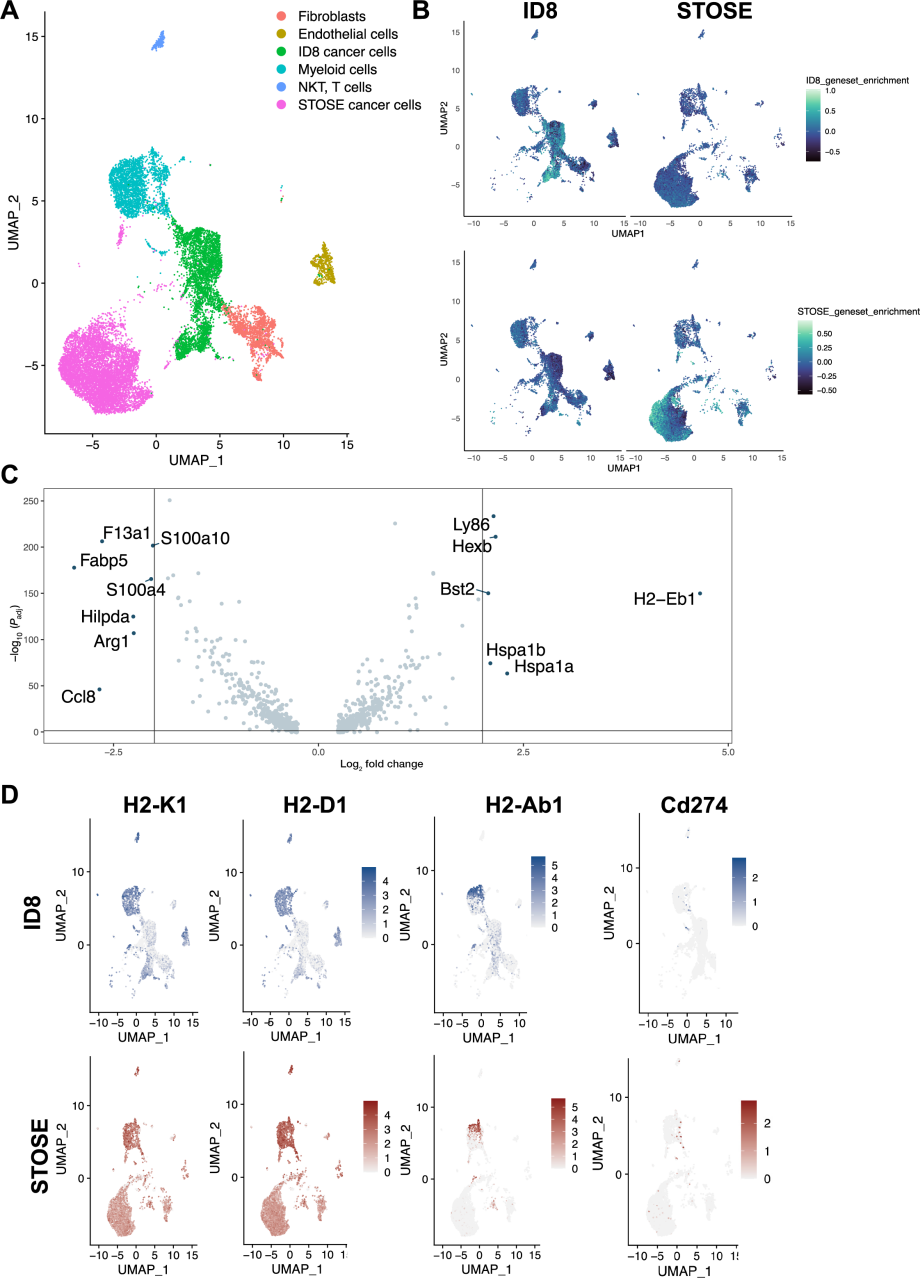
scRNA-seq reveals high heterogeneity among STOSE and ID8 cancer cells. **A,** scRNA-seq UMAP figures depicting cell clusters found in orthotopic ID8-WT (left) and STOSE (right) tumors at endpoint. Tumors were integrated into a gene expression matrix containing expression values of 17,853 cells and 20,091 genes, using Seurat. **B,** UMAP plot showing enrichment of individual cancer cells for gene-sets generated for ID8 and STOSE cancer cell identity (Supplementary Table S4). **C,** Volcano plot showing the most differentially expressed genes (DGE) between myeloid cell populations of orthotopic ID8-WT (left) and STOSE-WT (right) tumors beyond log_2_ fold-change threshold of 2. DGEs calculated using MAST test comparing ID8 and STOSE myeloid cells head-to-head. **D,** UMAPs representing expression of MHC-I haplotypes *H2-K1* and *H2-D1*, MHC-II haplotype *H2-Ab1*, and *Cd274* in ID8-WT and STOSE orthotopic tumors. Heatmap displays the level of expression in each cell type cluster (as identified in A) in ID8-WT (blue) and STOSE (red) samples.

To fully elucidate the tumor immune composition of syngeneic models, we first assessed how models of similar origins but different strains compare. Orthotopic tumors from OSE-derived ID8-WT from C57BL/6 mice (16) and STOSE from FVB/N mice (20) were collected close to endpoint and pooled for scRNA-seq analysis. Clustering (Fig. 1A) identified five major populations, including cancer, endothelial cells, fibroblasts, and myeloid and lymphocytic immune cells. Although both ID8 and STOSE cancer cells originated from the OSE, and both resulting tumor types expressed markers of HGSC such as WT1, overall they displayed distinct transcriptional profiles (Fig. 1B; Supplementary Table S4; Supplementary Fig. S1A); for example, *Amhr2*, *Star*, and *Sox9* were preferentially expressed in ID8-WT and *Ccl2*, *Il33*, and *Col1a3* were expressed in STOSE (Supplementary Fig. S1B). There were few cells captured in the lymphoid compartment [including T, natural killer (NK), and B cells] which prevented further analysis. The myeloid compartments of the two models were distinct (Fig. 1C). We generated signatures of M0/M1/M2 phenotypes and found that STOSE tumors were enriched with M2 TAMs [F13a1 (27), Fabp5 (28), S100a4 and Arg1 (26); Supplementary Fig. S2; Supplementary Table S5; ref. 26]. The immunogenicity of the cancer cells was explored by analyzing MHC-I, -II, and PD-L1 transcripts. H2-K1 and H2-D1 expression (MHC-I) was very low in ID8 cells but was more evident in immune and endothelial cell clusters (Fig. 1D). In contrast, STOSE tumors had strong MHC-I expression in most cellular components, including cancer cells. Some ID8 cancer cells expressed low amounts of H2-Ab1 (MHC-II), but in STOSE tumors, it was attributed only to the immune population. Cd274 (PD-L1) was poorly expressed in the cancer cells and immune populations of both tumor types.

Taken together, the scRNA-seq analysis suggests that STOSE tumors are preferentially infiltrated by immunosuppressive M2 TAM. Although the ID8 and STOSE share the same cellular origin, they have very different MHC-I expression profiles, indicating different capabilities to present tumor-associated antigens (TAA). These differences focused our attention on the immunogenicity and immune infiltration in the syngeneic models.

### ID8 Models Have Lost MHC-I Expression and do not Confer Antitumoral Protection When Administered as a Cellular Vaccine

**FIGURE 2:**
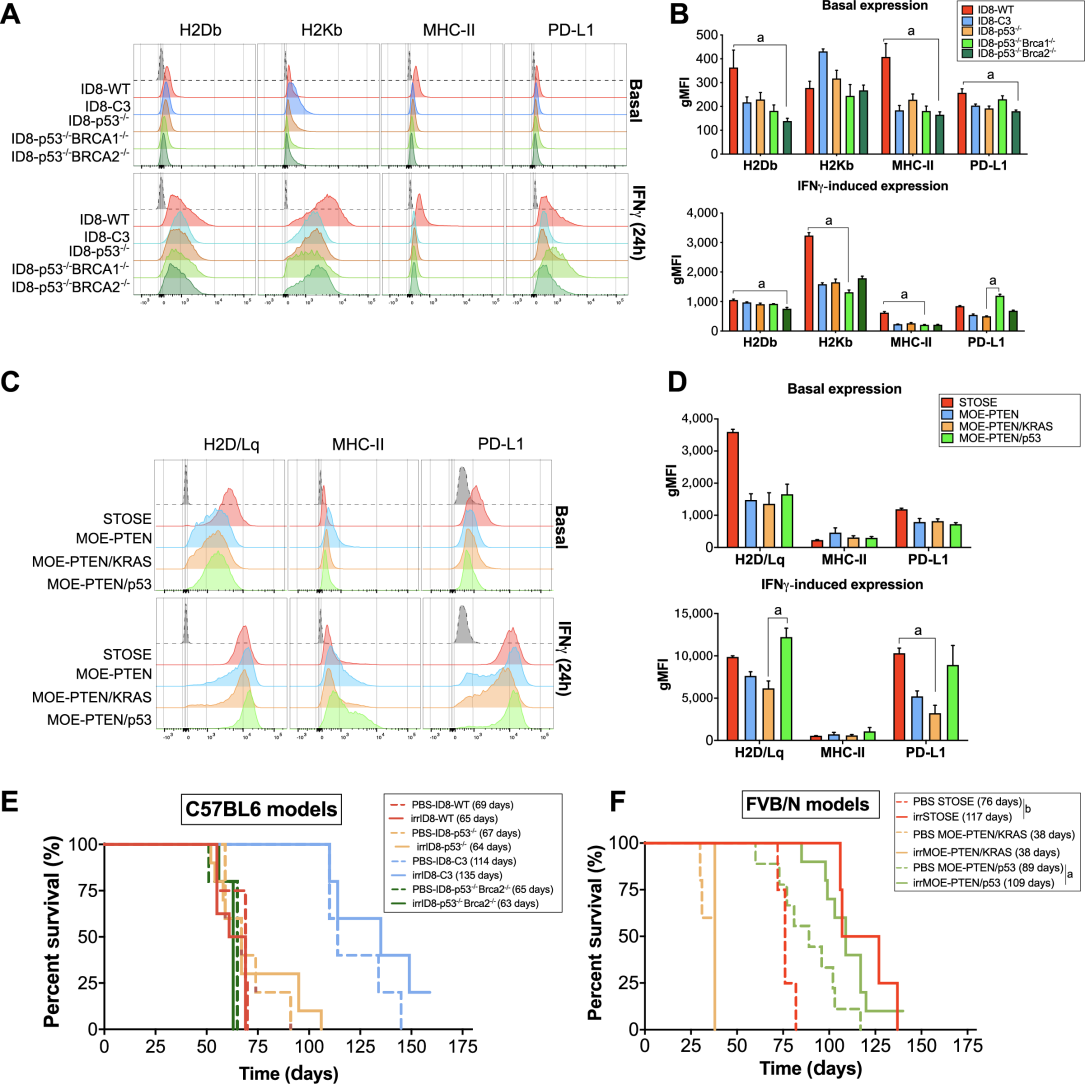
Ovarian cancer cell lines originating from FVB/N mice are more immunogenic. **A–D,***In vitro* expression of MHC-I, MHC-II, and PD-L1 on ID8 and its derivative cell lines. Flow cytometry on single, viable cells. Fluorescence minus one (FMO) are depicted in gray. **A,** Histograms represent the mean fluorescence intensity (MFI) of each marker at basal levels (top) and after IFNγ treatment for 24 hours (bottom). **B,** IFNγ induction for the proteins of interest was quantified and depicted as geoMFI (*n* = 3). Significance was determined by Kruskal–Wallis test (a, *P* < 0.05). **C,** Histograms showing basal expression of MHC-I haplotypes, MHC-II and PD-L1 on STOSE and MOE cell lines, which are further induced by IFNγ treatment. gMFI quantification shown in **D**. **E,** Survival Kaplan–Meier plots of ovarian tumor-bearing mice from C57BL/6 strain and treated with syngeneic cellular vaccines. 5 × 10^6^ cancer cells were irradiated (100 Gy) and injected intraperitoneally two weeks after injection of the same number of viable cells. PBS was injected as a control. Curves represent mice as follows: *n* = 5 PBS for each model, *n* = 8 ID8-WT, *n* = 5 ID8-C3, *n* = 10 ID8-p53^−/−^, and *n* = 5 ID8-p53^−/−^Brca2^−/−^. **F,** Survival Kaplan–Meier plots of FVB/N-derived ovarian cancer cell lines, treated with syngeneic cellular vaccines (irradiated at 60 Gy) as in **E**. Curves represent mice as follows: *n* = 4–10 PBS for each model, *n* = 4 STOSE, *n* = 4 MOE-PTEN/KRAS, *n* = 10 MOE-PTEN/P53. Log-rank (Mantel–Cox; a, *P* < 0.05; b, *P* < 0.01).

Tumor antigen presentation is essential to activate adaptive immune responses which are fundamental for antitumoral immunity. The striking differences in MHC expression in ID8 and STOSE cancer cells prompted us to further investigate the immunogenicity of the syngeneic models. First, we screened nine EOC cell lines, including those of oviductal origin (22) bearing *Trp53* mutation, *PTEN* suppression, and constitutive KRAS activation, and ID8-derived cell lines (18). Using flow cytometry, all ID8-derived cell lines displayed very low levels of MHC-I, MHC-II, and PD-L1 expression (Fig. 2A and B, top), while those sharing the FVB/N background maintained strong MHC-I expression regardless of their genotype or tissue of origin (Fig. 2C and D, top). To determine whether the low MHC-I expression in ID8 cells was reversible, they were treated with IFNγ for 24 hours. As shown in Fig. 2A and B (bottom), IFNγ treatment enhanced MHC-I, PD-L1, and to a lesser extent MHC-II expression in ID8 derivatives, with ID8-p53^−/−^Brca1^−/−^ cells displaying the highest induction of PD-L1. IFNγ also increased expression of these molecules in the FVB/N cell lines (Fig. 2C and D, bottom), with MOE-PTEN/p53 showing the strongest induction of MHC-I expression. PD-L1 levels were most responsive to treatment in STOSE and MOE-PTEN/p53 cells. FVB/N-derived cell lines expressed 5-fold higher levels of PD-L1 compared with C57BL/6 models. qPCR analysis confirmed that transcript levels were consistent with protein levels (Supplementary Fig. S3), suggesting that the lost MHC-I expression in the ID8-derived cell lines is reversible and may be an immune evasion mechanism exploited by these cells.

To test the immunogenicity of the syngeneic models *in vivo*, mice were injected intraperitoneally with viable cells and 14 days later, received lethally irradiated cells intraperitoneally. Mice injected with irradiated ID8 derivatives reached endpoint at a similar time as control mice (Fig. 2E), corroborating the poor immunogenicity of these cell lines. Conversely, inoculation of irradiated STOSE and MOE-PTEN/p53 significantly increased median survival of tumor-bearing mice compared with the control groups (Fig. 2F). Despite the fact that all the cell lines can significantly increase MHC-I, MHC-II, and PD-L1 expression under IFNγ treatment, only the STOSE and MOE-PTEN/p53 are immunogenic, evident by the increased survival of tumor-bearing mice when the cells are administered as a cellular vaccine.

### STOSE and MOE-PTEN/KRAS Orthotopic Tumors are Preferentially Infiltrated by Myeloid-Like Immune Cells

To further characterize the syngeneic models, orthotopic tumors were generated by injecting 0.15 × 10^6^ cells under the ovarian bursa. Each cell line resulted in mice reaching median survival similar to those observed when cells were injected at a higher number (5 × 10^6^ cells) into the peritoneal cavity (Supplementary Table S6). MOE-PTEN and MOE-PTEN/p53 were the exceptions, with lower frequencies of orthotopic tumor development (6% and 15%, respectively) even after 300 days. Comparing mouse, spleen, and tumor masses (Supplementary Fig. S4), we observed STOSE and MOE-PTEN/KRAS cells tended to generate larger orthotopic tumors (Supplementary Fig. S4C), while ID8-derived models generated more extensive peritoneal disease (Supplementary Fig. S4D) and ID8-WT mice had higher ascites accumulation (Supplementary Fig. S4E). STOSE and MOE-PTEN/KRAS models progressed quickly in the last week before humane endpoint, developing extensive peritoneal metastasis and approximately 4 mL of ascites. Histologically, tumors consisted of very dense epithelioid cells and increased nuclear atypia, with the STOSE model having frequent association with adipocytes (Supplementary Fig. S4F). MOE-PTEN/KRAS tumors consistently contained multiple small areas of necrosis (Supplementary Fig. S4G).

**FIGURE 3:**
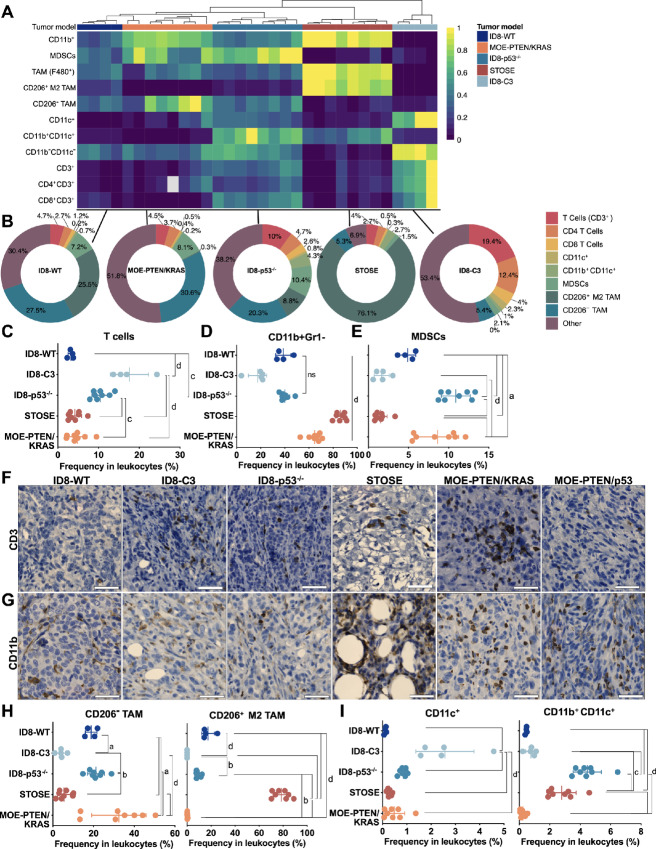
ID8-C3 and ID8-p53^−/−^ tumors recruit more T cells, while STOSE and MOE-PTEN/KRAS are more infiltrated by TAMs. **A,** Heatmap depicts normalized relative frequency of all the studied immune cell populations for all tumor models, determined by flow cytometry. White square is an omitted outlier sample. **B,** Pie charts showing relative distribution of main immune cell populations within each tumor type based on frequency (%) in CD45^+^ cell population. Other includes CD11b^−^CD11c^−^ and CD11b^+^F480^−^ populations. **C,** Percentage of CD3^+^ cells in leukocytes found in orthotopic tumor models as assessed by flow cytometry. Total frequency among leukocytes of CD11b^+^Gr1^−^ (**D**) and MDSCs (**E**). **F,** IHC detection of CD3^+^ cells in tumors showing CD3 stained clusters in MOE-PTEN/KRAS samples. See Supplementary Fig. S6A–S6C for quantification of cells/mm^2^ and cluster representation at a lower magnification. **G,** Representative images showing CD11b^+^ cells for all tumor models. Sections were counterstained with hematoxylin (blue) and positive cells (brown) were stained with DAB. Scale bars, 50 μm. See Supplementary Fig. S7B for quantification of cells/mm^2^. **H,** Frequencies of CD206^−^ TAMs and CD206^+^ M2 TAMs as well as CD11b^−^CD11c^+^ and CD11b^+^CD11c^+^ DCs as determined by flow cytometry (**I**). For flow cytometry analysis, cell frequencies were determined by discriminating doublets, dead cells, and CD3^+/−^ cells (see Supplementary Fig. S5). Each dot represents an orthotopic tumor. Mean values with SD are shown. Significance was determined by one-way ANOVA with Tukey *post test* comparing all models; ns, not significant; a, *P* < 0.05; b, *P* < 0.01; c, *P* < 0.001; d, *P* < 0.0001.

**FIGURE 4:**
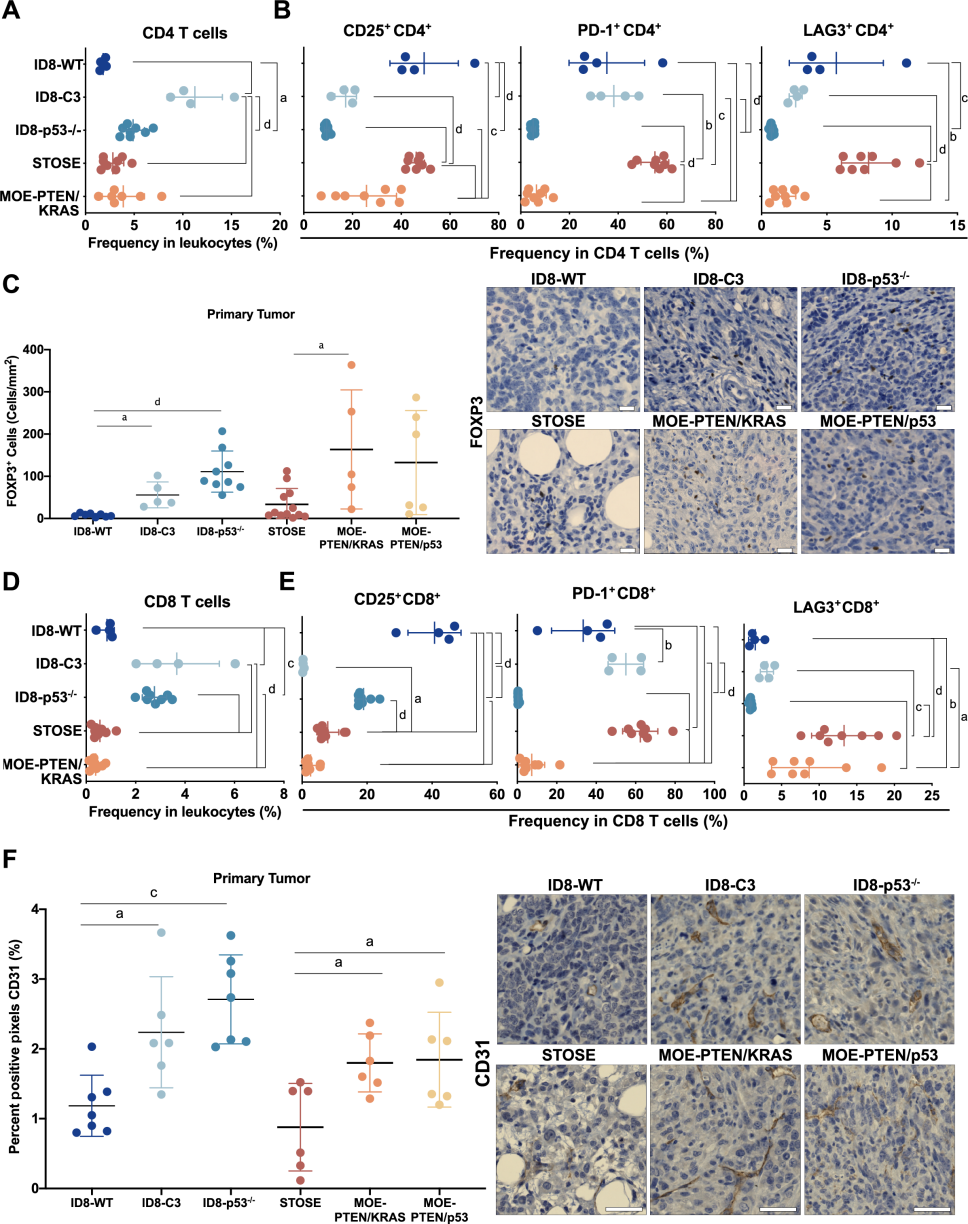
STOSE tumors have greater frequency of CD4 and CD8 T cells expressing exhaustion markers. Flow cytometry analysis showing the frequencies of CD4^+^-expressing cells (**A**) and CD25, PD-1, and LAG3 expression (**B**) among CD4^+^ T cells. **C,** Images representative of FOXP3^+^ staining in ID8-WT (*n* = 8), ID8-C3 (*n* = 6), ID8-p53^−/−^ (*n* = 9), STOSE (*n* = 12), MOE-PTEN/KRAS (*n* = 6), and MOE-PTEN/p53 (*n* = 6) samples. Cell counts (plotted as number of cells/mm^2^) were quantified using ImagePro Premier. **D** and **E,** Flow cytometry analysis showing the frequencies of CD8^+^ T cells (**D**) among leukocytes in each tumor model and CD25 (**E**), PD-1 and LAG3 expression among CD8^+^ T cells. For flow cytometry analysis, cell frequencies were determined by discriminating doublets, dead cells, CD45^−^, and CD3^+^ cells (see Supplementary Fig. S5A). Each dot represents an orthotopic tumor. Mean values with SD are shown. Significance was determined by one-way ANOVA with Tukey *post test* comparing all models; a, *P* < 0.05; b, *P* < 0.01; c, *P* < 0.001; d, *P* < 0.0001. **F,** Quantification (left) and IHC detection of endothelial cells by CD31 staining (right) in all tumor models. Data was quantified using Orbit Image analysis (% positive area). Each dot represents an orthotopic tumor. Mean values with SD are shown. Scale bars, 20 μm (FOXP3) and 50 μm (CD31). For IHC, sections were counterstained with hematoxylin (blue) and positive cells (brown) with DAB. Mean values with SD are shown. Significance was determined by one-way ANOVA within C57BL/6 or FVB/N models with Tukey *post test* or a two-tailed Student *t* test (comparing ID8 and STOSE); a, *P* < 0.05; d, *P* < 0.0001.

**FIGURE 5:**
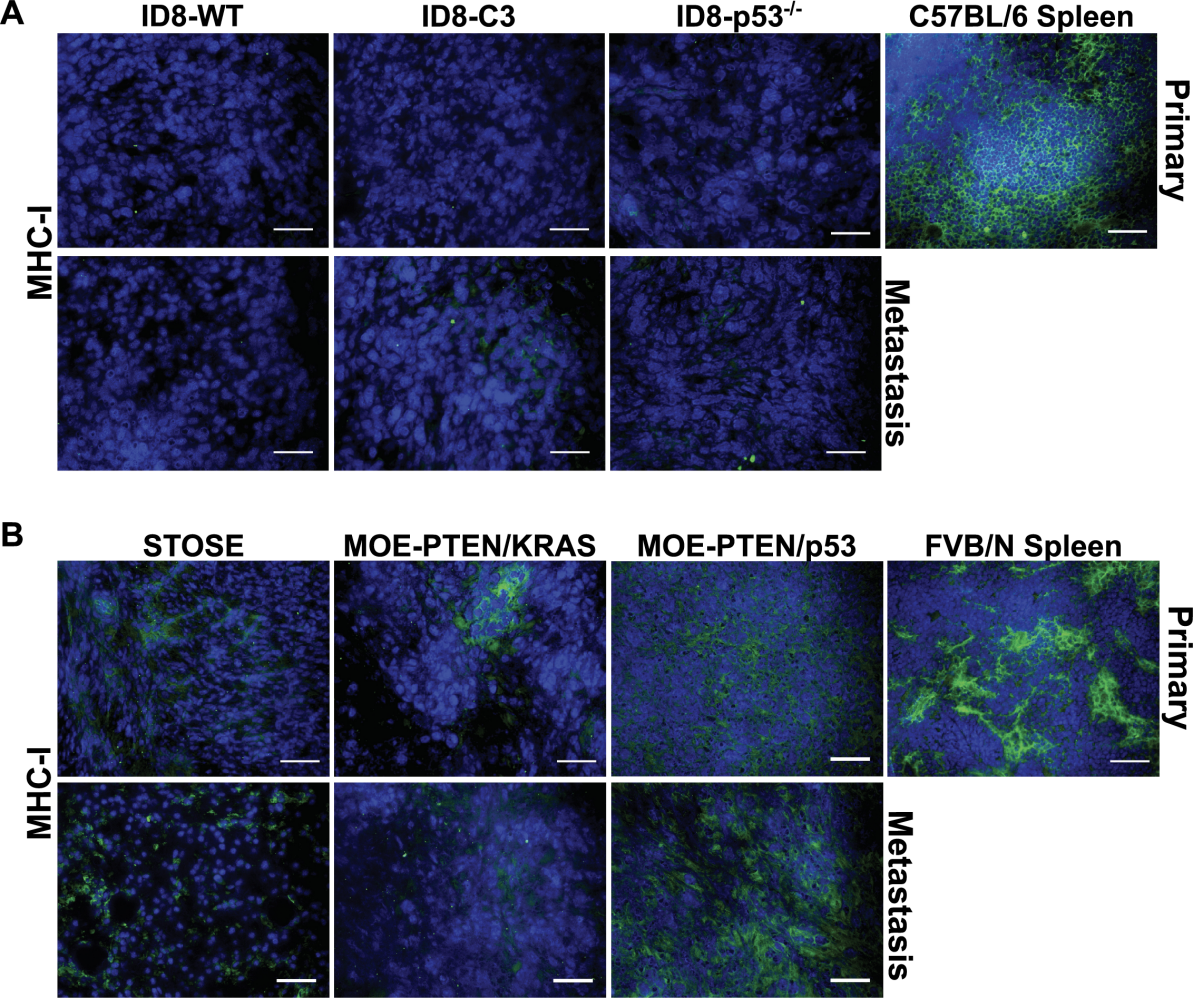
Primary and metastatic tumors from ID8-derived cancer cells do not express MHC-I *in vivo*, while STOSE and MOE models do. **A,** Immunofluorescence depicting MHC-I (green) and nuclei (Hoechst) on primary (top) and metastatic (bottom) tumors showing little to no expression of MHC-I. C57BL/6 spleen was used as a positive control. Images are representative of *n* = 3 primary/metastasis for each model. Scale bars, 50 μm. **B,** Primary and metastatic tumors derived from the FVB/N models retained MHC-I expression (green) as shown by immunofluorescence. Scale bars, 50 μm.

**FIGURE 6:**
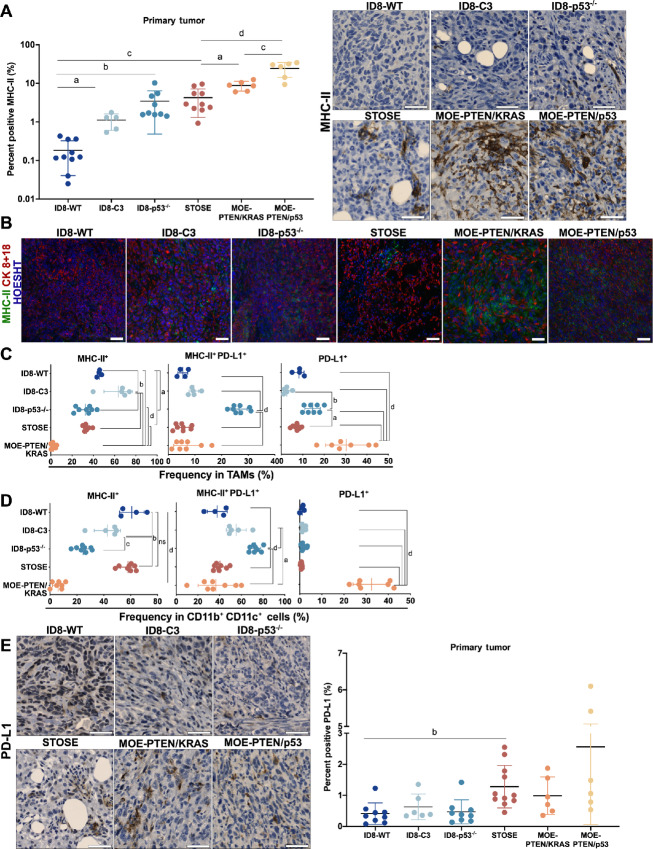
The TME of FVB/N models is characterized by strong MHC-II expression. **A,** IHC detection (right) and quantification of MHC-II^+^ cells (left) in all tumor models. Sections were counterstained with hematoxylin (blue) and positive cells with DAB (brown). Images are representative of tumors for each model as follows: ID8-WT (*n* = 10), ID8-C3 (*n* = 6), ID8-p53^−/−^ (*n* = 9), STOSE (*n* = 10), MOE-PTEN/KRAS (*n* = 6), MOE-PTEN/p53 (*n* = 6) samples. Scale bars, 50 μm. Percent positive MHC-II (%) areas of the tumor were quantified using Orbit Image analysis. **B,** Immunofluorescence depicting MHC-II (green), Cytokeratin 8+18 (red), and nuclei (Hoechst) on primary tumors. Images are representative of *n* = 3 primary tumors for each model. Scale bars, 50 μm. **C** and **D,** MHC-II and PD-L1 expression in TAMs (**C**) and CD11b^+^CD11c^+^ (**D**) subsets found in the TME, assessed by flow cytometry. Mean values with SEM are shown for each tumor model. Significance was determined by one-way ANOVA with Tukey *post test* comparing all models; ns, not significant; a, *P* < 0.05; b, *P* < 0.01; c, *P* < 0.001; d, *P* < 0.0001. **E,** Quantification (right) and IHC detection (left) of PD-L1 expression in all tumor models. Sections were counterstained with hematoxylin (blue) and positive cells with DAB (brown). Images are representative of ID8-WT (*n* = 9), ID8-C3 (*n* = 6), ID8-p53^−/−^ (*n* = 9), STOSE (*n* = 12), MOE-PTEN/KRAS (*n* = 6), MOE-PTEN/p53 (*n* = 6) tumors. Scale bars, 50 μm. Percent positive PD-L1 (%) areas of the tumor were quantified using Orbit Image analysis. Mean values with SD are shown for each tumor model. Significance was determined by one-way ANOVA within C57BL/6 or FVB/N models with Tukey *post test* or a two-tailed Student *t* test (comparing ID8 and STOSE).

To compare the immune cell composition, orthotopic tumors were analyzed by flow cytometry following the gating strategy shown in Supplementary Fig. S5 and, in parallel, by IHC staining. We performed a conventional screen for T cells, MDSCs, TAMs, monocytes, and DCs (Fig. 3), as well as additional markers to assess their potential functional phenotype (Figs. 4–6; Supplementary Figs. S6–S11). Data were normalized for each main population and summarized in a heatmap with unsupervised hierarchical clustering (Fig. 3A). The data cluster samples from each model together, highlighting the distinct TME of each model. Overall, MOE-PTEN/KRAS and STOSE are more infiltrated by myeloid populations, while the ID8-p53^−/−^ and ID8-C3 tumors are more infiltrated by T cells (Fig. 3B). Notably, the ID8-C3 (CRISPR control) is not equivalent to the ID8-WT.

ID8-WT tumors possess the “coldest/immune desert” profile with very poor T-cell recruitment, while ID8-p53^−/−^ and ID8-C3 tumors have the highest frequencies of T cells (>10% of all leukocytes; Fig. 3C). Quantification of these cells by IHC supported the findings by flow cytometry and scRNA-seq, with ID8-WT and STOSE tumors being poorly infiltrated by CD3^+^ cells (Fig. 3F; Supplementary Fig. S6A), despite the fact that the STOSE model was able to confer antitumoral protection when administered as a cellular vaccine (Fig. 2F). Due to the frequent rejection of tumors in the orthotopic MOE-PTEN/p53 model, the TME was only evaluated by IHC, which found CD3^+^ cells in similar abundance as in MOE-PTEN/KRAS tumors, with slightly higher CD3^+^ cells/mm^2^ compared with STOSE (Fig. 3F; Supplementary Fig. S6A). Interestingly, while CD3^+^ cells were randomly distributed in most tumors, they formed distinct clusters or areas with intense staining in MOE-PTEN/KRAS tumors (Fig. 3F; Supplementary Fig. S6B), although necrotic areas were largely devoid of T cells. Quantification of CD3^+^ staining in primary versus metastatic lesions revealed that ID8-WT metastases had proportionally more CD3^+^ cells, while metastases from STOSE tumors had fewer CD3^+^ cells (Supplementary Fig. S6C).

Detailed analyses of the myeloid-like populations are summarized in Fig. 3A and B and Supplementary Fig. S7A. Further analysis of the CD11b^+^ population with Gr1 coexpression identified MDSCs (CD11b+Gr1^hi^), a heterogeneous population of polymorphonuclear MDSCs (PMN-MDSC) and CD11b+Gr^−/low^ cells including monocytes, macrophages, and DCs (29). MOE-PTEN/KRAS and STOSE were the most infiltrated by CD11b^+^ cells (CD11b^+^CD11c-Gr1^low^) representing more than 75% of all leukocytes (Fig. 3D) and in STOSE tumors almost 1,000 cells/mm^2^ (Fig. 3G; Supplementary Fig. S7B and S7C). Although the frequency of CD11b^+^ cells was high by flow in MOE-PTEN/KRAS, IHC quantification showed similar abundance of CD11b^+^ cells to ID8 models, likely due to large areas of necrotic tissue with few immune cells present (Supplementary Fig. S3D). No difference was observed in the prevalence of CD11b^+^ cells in primary tumors versus metastases (Supplementary Fig. S7B). MDSCs were dominant in ID8-p53^−/−^ and MOE-PTEN/KRAS, and to a lesser extent in ID8-WT (>10%, ∼7%, and <5% among all leukocytes; Fig. 3E).

Analysis of the CD11b^+^ population with TAM markers, showed that STOSE tumors were the most infiltrated by M2 CD206^+^ F4/80^+^ TAM (80% of all leukocytes) constituting the majority of immune cells in these tumors (Fig. 3B) and confirming scRNA-seq findings (Fig. 1C; Supplementary Fig. S2). In contrast, MOE-PTEN/KRAS tumors were infiltrated predominantly by CD206^−^ TAMs (Fig. 3B–H). As previously shown by Walton and colleagues, ID8-p53^−/−^ contained more TAMs (CD11b^+^CD11c-F4/80^+^) compared with ID8-C3 tumors (ref. 18; Fig. 3H; Supplementary Fig. S7C). Interestingly, ID8-C3 differed from all other models by the infiltration of myeloid-like cells composed mainly of CD11b^−^CD11c^+^ (Fig. 3B and I), which could represent subsets of DCs (30) and CD11b^−^CD11c^−^ (Supplementary Fig. S7D; ∼3 and ∼40%, respectively, of all leukocytes). All the other tumors contained less than 1% of CD11c^+^ cells. Finally, ID8-p53^−/−^ and STOSE were significantly infiltrated by CD11b^+^CD11c^+^ cells, which includes conventional DC2s (cDC2) that are mainly recognized by the induction of CD4^+^ T-cell immunity in cancer (ref. 31; Fig. 3I). Taken together, these findings suggest that STOSE and MOE-PTEN/KRAS tumors are preferentially infiltrated by CD11b^+^ myeloid-like immune cells while most of the ID8-derived models possess greater capability to recruit T cells.

### STOSE Tumors Contain T Cells Displaying High Expression of Exhaustion Markers

To investigate the T-cell compartment, we examined the activation/exhaustion markers CD25, PD-1, and LAG3 on CD4 and CD8 T-cell subsets (Supplementary Fig. S6D) by flow cytometry, and FOXP3^+^ expression by IHC, suggestive of Tregs. ID8-C3 tumors contained the highest frequencies of CD4^+^ T cells (∼10% of all tumor-infiltrating leukocytes; Fig. 4A). CD4^+^ T cells from ID8-p53^−/−^ tumors displayed very poor expression of CD25, PD-1, and LAG3 compared with all other models (Fig. 4B), while STOSE and ID8-WT, the most poorly infiltrated by T cells, expressed more CD25, PD-1, and LAG3. ID8-C3 and ID8-p53^−/−^ tumors contained more FOXP3^+^ cells/mm^2^ compared with ID8-WT (Fig. 4C), corresponding to trends seen for CD3 infiltration. Considerable variability was found in the FOXP3^+^ populations in the two MOE models, largely due to the presence of necrotic areas with few immune cells (Supplementary Fig. S4G). When comparing primary to metastatic tumors, ID8-WT metastatic tumors had a higher abundance of FOXP3^+^ cells (Supplementary Fig. S6E).

CD8^+^ T cells comprised more than 2% of the leukocytic population only in the ID8-C3 and ID8-p53^−/−^ models (Fig 4D). Remarkably, the model containing the highest CD4/CD8 T-cell ratio was MOE-PTEN/KRAS with approximately 7 CD4^+^ T cells for each CD8^+^ T cell, unveiling a potential shift to CD4 Th2 response as an immune evasion mechanism (Supplementary Fig S6F). ID8-WT tumors contained the highest frequency of CD25^+^CD8^+^ T cells (>40% among all CD8^+^ T cells) and PD-1^+^CD8^+^ T cells (Fig. 4E). ID8-C3 tumors also possessed high frequencies of PD-1^+^CD8^+^ T cells. Interestingly, CD8^+^ T cells in ID8-p53^−/−^ tumors expressed CD25 but not exhaustion markers, PD-1 and LAG3. STOSE and MOE-PTEN/KRAS had fewer CD25^+^CD8^+^ T cells and more LAG3^+^CD8^+^ T cells (>10%), while STOSE tumors contained the highest frequency of PD-1^+^ CD8^+^ T cells (∼60%).

To determine whether tumor vasculature might be associated with differences in immune cell infiltration, we stained for CD31 by IHC and found less CD31^+^ area in ID8-WT and STOSE tumors compared with the other models (Fig. 4F), which interestingly corresponded to tumors most poorly infiltrated by T cells (Fig. 3C). Conversely, ID8-C3, ID8-p53, MOE-PTEN/KRAS, and MOE-PTEN/p53 tumors displayed more CD31^+^ area, allowing for potentially more immune infiltration and corresponding to greater numbers of CD3^+^ cells/mm^2^ (Supplementary Fig S6A).

Collectively, our findings suggest that ID8-WT and STOSE tumors, having the lowest CD31 expression, were the most poorly infiltrated by T cells, these having higher expression of activation/exhaustion markers which may indicate a more impaired phenotype. Moreover, ID8-p53^−/−^ and MOE-PTEN/KRAS tumors contained more FOXP3^+^ cells, potentially Tregs, but only the MOE-PTEN/KRAS model had a higher ratio of CD4/CD8, approximately 25% CD25^+^CD4^+^ T cells (potentially including Tregs) and low CD25^+^ and PD-1^+^ expression on CD8^+^ T cells that suggests a lack of T-cell activation in the TME.

### Stromal Cells of Tumors Derived from Fallopian Tube Epithelium Express High Levels of MHC-II

Given that TAA presentation and response to immunotherapy are closely linked to MHC and PD-L1 expression (32, 33), we next characterized their expression in tumors. By immunofluorescence (IF), we found ID8-derived orthotopic tumors expressed very low levels of MHC-I, similar to the cell lines *in vitro*, while STOSE and MOE-derived tumors maintained their positivity *in vivo* (Fig. 5). Interestingly, comparable MHC-I expression was observed in the metastatic lesions for each tumor model (Fig. 5). Staining for MHC-II by IHC showed ID8-p53^−/−^ and ID8-C3 tumors had more MHC-II^+^ area compared with ID8-WT, while STOSE and MOE models displayed higher MHC-II^+^ area that was most abundant in MOE-PTEN/KRAS and MOE-PTEN/p53 tumors (∼10% and ∼25% positive area, respectively; Fig. 6A). As observed with CD3 staining (Supplementary Fig. S6B), MHC-II staining was found in clusters (Supplementary Fig. S8A) in MOE-PTEN/KRAS sections, displaying characteristics typical of immune cell clusters. Interestingly, metastases from ID8-WT tumors had higher MHC-II^+^ area compared with their primary tumors, while all other models were roughly equivalent between primary and metastatic lesions (Supplementary Fig. S8B). To further explore the strong MHC-II expression found in the MOE models, double IF staining for MHC-II and CK8^+^18 was performed. A lack of costaining of MHC-II with epithelial cancer cells (Fig. 6B) raised the question of the main source of MHC-II. Flow cytometry analysis showed a low frequency of MHC-II positive immune cells in the myeloid compartment (Fig. 6C and D), and low levels of expression (Supplementary Fig. S8C) relative to the other models. Using serial sections, MHC-II staining was shown to be in excess of the staining for CD11b, suggesting that CD11b^−^ cells also express MHC-II (Supplementary Fig. S8D). Therefore, the high expression of MHC-II in the TME of MOE models appears to be primarily associated with stromal components other than immune cells.

We next examined MHC-II and PD-L1 expression in the myeloid populations found most frequently in tumors, TAMs and cDC2s. While MOE-PTEN/KRAS tumors had the lowest frequency of MHC-II^+^ TAMs and cDC2s, they had the highest frequency (∼30%) of PD-L1^+^ TAMs and cDC2s (Fig. 6C and D). Interestingly, ID8-p53^−/−^ tumors contained the highest frequency of double positive MHC-II and PD-L1 TAMs and cDC2s. MHC-II expression was more pronounced on CD206^+^ M2 TAMs and DCs derived from the ID8-C3 model, and to a lesser extent from ID8-WT tumors (Supplementary Fig. S8C). TAMs and cDC2s in STOSE and ID8-WT were largely MHC-II positive, but had the lowest frequency (Fig. 6C–E) and expression levels (Supplementary Fig. S9A) of PD-L1. Analysis of PD-L1 expression on CD45-negative cells (Supplementary Fig. S9B) showed that STOSE tumors had high levels. This was confirmed by IHC, where STOSE and MOE tumors had more PD-L1^+^ area (Fig. 6E). STOSE metastases had a significantly lower percent positive PD-L1 expression compared with the primary tumors, while metastases from ID8-derived models had on average approximately 0.5% positive staining. The STOSE and MOE models had approximately 1.5% positive staining, but these models also had some metastatic samples with much higher staining of PD-L1, ranging between 5% and 20% (Supplementary Fig S9C).

Taken together, we found that ID8-derived tumors have lost their capability to express MHC-I *in vivo* while STOSE and MOE-PTEN/KRAS-derived models maintained their expression. Moreover, the MOE-PTEN/KRAS model consists of highly immunosuppressive TAMs and cDC2 cells with high PD-L1 expression, unique to this model. This model also has strong MHC-II expression in the tumor niche, similar to the MOE-PTEN/p53^−/−^ tumor model.

### The Immune Profile of Ascites from MOE-PTEN/KRAS Mice Mirrors the TME Composition

In ovarian cancer, peritoneal ascites adds to tumor burden and plays a major role in influencing therapeutic outcome. We therefore determined the immune profile of the spleen and ascites at the time when orthotopic tumors were collected and compared the frequencies of immune subsets to tumor-naïve tissues for each mouse strain. Splenocytes subsets were similar to tumor-naive controls (Supplementary Fig S10A) with only MOE-PTEN/KRAS showing different proportions of immune cells compared with FVB/N control spleens. Higher infiltration of CD11b^+^ cells was found in MOE-PTEN/KRAS (>10% from all leukocytes), and within this population, TAMs were the most prominent (Supplementary Fig. S10B).

**FIGURE 7:**
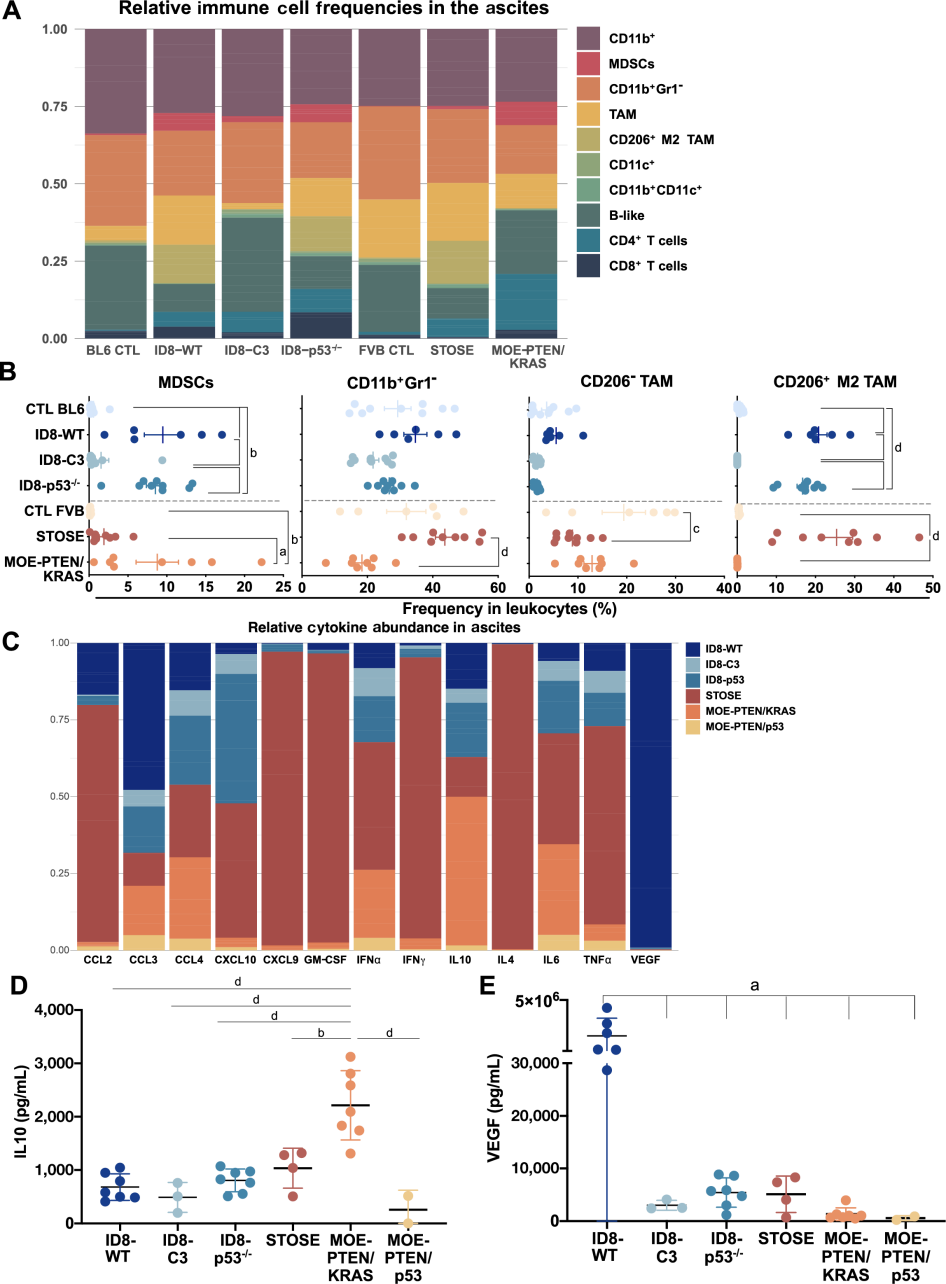
The ascites immune composition and chemo/cytokine network highlights the heterogeneity of the murine orthotopic ovarian cancer models. **A,** Stacked-bar figures showing the relative frequencies of several immune populations found in the ascites. **B,** Significantly different frequencies of the myeloid-like compartment present in the ascites assessed by flow cytometry. Age-matched tumor-naive C57BL/6 and FVB/N mice were included as controls. Cells were analyzed as shown in Supplementary Fig. S5A. Each dot represents a single sample derived from an orthotopic tumor-bearing or control mouse. Mean values with SEM are shown for each tumor model. Significance was determined by one-way ANOVA with Tukey *post test* comparing all models; ns, not significant; a, *P* < 0.05; b, *P* < 0.01; c, *P* < 0.001; d, *P* < 0.0001. **C,** Stacked-bar figures showing the relative abundance of cytokines and chemokines found in the ascites fluid derived from orthotopic tumor-bearing mice. ID8-WT (*n* = 7), ID8-C3 (*n* = 3), ID8-p53^−/−^ (*n* = 7), STOSE (*n* = 4), MOE-PTEN/KRAS (*n* = 7), MOE-PTEN/p53 (*n* = 2). IL10 (**D**) and VEGF abundance (**E**; pg/mL) in the ascites fluid of each tumor model. Chemo/cytokines were measured by LEGENDplex Mouse Cytokine Release Syndrome Panel (13-plex) Multi-Analyte Flow Assay. Each dot represents a single sample derived from the supernatant of ascites from tumor-bearing mice. Mean values with SD are shown for each tumor model. Significance was determined by one-way ANOVA with Tukey *post test* comparing all models; a, *P* < 0.05; b, *P* < 0.01; d, *P* < 0.0001.

In the ascites, the immune subsets present in the ID8-C3 model were similar to the C57BL/6 control sample, with a higher proportion of CD4^+^ T cells, while ID8-WT and ID8-p53^−/−^ were more similar (Fig. 7A), preferentially recruiting MDSCs and CD206^+^ M2 TAMs (∼10 and >20% of all leukocytes, respectively; Fig. 7B). Consistent with the results from the tumors, MOE-PTEN/KRAS ascites preferentially attracted more CD4^+^ T cells (Fig. 7A) and MDSCs (∼10% of all leukocytes; Fig. 7B). STOSE ascites also displayed a similar profile to the tumors with more infiltration by CD11b^+^Gr1^−^ and CD206^+^ M2 TAMs, representing >40% and 25% of all immune cells in the ascites fluid, respectively, while CD206^−^ TAM proportions were similar between models (Fig. 7B). Collectively, these findings indicate that the immune profile found in the TME was replicated in the ascites and spleen, with higher frequencies of MDSCs and CD4^+^ T cells in the MOE-PTEN/KRAS model, and higher TAM infiltration in the STOSE model.

We next assessed whether the MHC-II and PD-L1 expression found in TAMs and cDC2s in the ascites reflects their abundance in the tumors. In TAMs (Supplementary Fig. S11A), the frequency of MHC-II^+^ cells was higher only in ID8-C3 ascites while all other models had similar percentages. ID8-p53^−/−^ ascites differed significantly by their increased content of MHC-II^+^PD-L1^+^ TAMs. We observed more divergence in the proportion of PD-L1^+^ TAMs with more in ID8-WT, ID8-p53^−/−^, and STOSE ascites. MHC-II^+^ cDC2s were significantly lower in MOE-PTEN/KRAS samples, while being higher in ID8-WT ascites, and PD-L1^+^ cDC2s were found at highest frequencies in the ID8-p53^−/−^ ascites (Supplementary Fig. S11B). Finally, a screening of total PD-L1 expression on myeloid-like cells in the ascites revealed high PD-L1 expression in the TAMs from ID8-p53^−/−^ ascites and similarly elevated PD-L1 expression in the cDC2s in ID8-p53^−/−^ and MOE-PTEN/KRAS ascites, and to a lesser extent in the CD11c^+^ population (Supplementary Fig. S11C). Analysis of other nonimmune (CD45-) cells in the ascites found that, as observed in CD45^+^ populations, ID8-C3 ascites contained the highest MHC-II expression, in contrast to the MOE-PTEN/KRAS model which poorly expressed MHC-II on CD45^−^ cells (Supplementary Fig. S11D, left). Similar analysis of PD-L1 in other ascites cells identified an abundance of PD-L1^+^ cells in both ID8-p53^−/−^ and STOSE tumors (Supplementary Fig. S11D, right). In sum, the main source of PD-L1 expression in the ascitic immune compartment comes from TAMs and cDC2s found mainly in ID8-p53^−/−^ and MOE-PTEN/KRAS samples, revealing a more highly immunosuppressive TME.

Finally, to identify the cytokines and chemokines associated with immune cell recruitment, we used multiplexed cytokine assays to determine their abundance in ascites fluid (Supplementary Table S7) and plasma (Supplementary Table S8). Chemo/cytokines were not found in great abundance in the plasma, with only a few individual samples containing high levels, and no significant differences were observed between the models or between the tumor-bearing mice and controls (Supplementary Fig S12A). In ascites, 10 of 13 chemo/cytokines were significantly more abundant in the STOSE samples (Fig. 7C). Many, such as CCL2, CCL4, CXCL9, and CXCL10, likely contributed to the infiltration and positive feedback signaling of TAMs and MDSCs into the tumors and ascites of this model (7). MOE-PTEN/KRAS ascites contained the highest concentration of IL10, supporting the high prevalence of potentially tolerogenic cDC2s (Fig. 7D). VEGF was found at highest abundance in the ID8-WT model, likely explaining the poor T-cell infiltration and the high ascites accumulation particular to this model (Figs. 3C and 7E; Supplementary Table S6) as observed in human ovarian cancers (7). ID8-C3 and ID8-p53^−/−^ ascites displayed similar levels of chemo/cytokines, but differed from the ID8-WT ascites for IFNγ and CCL2 production (Supplementary Fig. S12B and S12C), and showed similar trends for TNFα and IL6 production (Supplementary Table S7). ID8-p53^−/−^ ascites fluid contained the highest concentration of CXCL10 among all C57BL/6 models (Supplementary Fig. S12D), which potentially can be produced by CD11c^+^ DCs, enabling a higher recruitment of CD8^+^ T cells to the ascites (Fig. 7A). Finally, our main findings were correlated with the scRNA-seq data from ID8-WT and STOSE orthotopic tumors, supporting *Vegfa* being highly expressed in ID8-WT tumors, and *Ccl2* in STOSE tumors (Supplementary Fig. S13). In addition, *Il18, Tnf, Ptgs2, Cd47, Cxcl1*, and *Csf1* were more highly expressed in STOSE tumors, including in the cancer cells, supporting the influence of TAMs in these tumors. Expression of *Ccl4* and *Il1b* found in immune cells was similar in ID8 and STOSE tumors. Taken together, chemokine/cytokines were profoundly more abundant in STOSE ascites fluid and their abundance correlated with the recruitment of CD206^+^ M2 TAMs found in the TME and peritoneal cavity of the STOSE model.

## Discussion

In this study, we extensively characterized the immunogenicity and TME at an advanced stage of EOC using models derived from different cells of origin (fallopian tube and ovarian epithelium), harboring mutations relevant to human disease, and from different mouse genetic backgrounds. The scope of our study did not cover NK, NK T, and B cells, which also play important roles in tumor immunity. We studied orthotopic tumors, but this approach was limited by the fact that some MOE models (MOE-PTEN/p53 and MOE-PTEN) did not grow well in the intrabursal cavity. This extensive characterization of the tumor immune composition of six syngeneic models of EOC will expand testing of immunotherapies for EOC.

In the initial characterization, cell lines and tumors from STOSE and MOE cells were found to be positive for MHC-I expression while all ID8-derived models expressed little to none. This suggests that cancer cells from the STOSE and MOE models could be more capable of presenting TAAs in the TME. Indeed, STOSE and MOE-PTEN/p53^−/−^ were able to induce antitumoral protection when used as a cellular vaccine. Therefore, ID8-derived tumors provide researchers with models to investigate treatments capable of overcoming MHC-I loss, associated with poor prognosis in EOC and limited response to immunotherapy in other cancer models (32).

Screening of the T-cell compartment revealed that ID8-WT and STOSE tumors were poorly infiltrated by T cells, mainly PD-1^+^. Poor T-cell recruitment may be attributed to low CD31^+^ cells, highlighting the poor vascularization in the TME (32). While ID8-WT primary tumors were poorly infiltrated, the metastases were abundant in CD3^+^ and FOXP3^+^ cells, and MHC-II^+^ areas. Chemo/cytokine ascites profiles were also different between the two models, potentially contributing to the recruitment of more CD11b^+^ cells in STOSE tumors. Analysis of these tumors by scRNA-seq indicated that STOSE tumors are preferentially infiltrated by immunosuppressive myeloid populations, which was confirmed by the abundance of CD206^+^ M2 TAMs detected by flow cytometry. These M2 TAMs, along with high PD-L1 expression, may trigger high expression of PD-1 and LAG3 on T cells. STOSE tumors appear rich in adipocytes, which could hinder T-cell function indirectly by perturbing T-cell metabolism or directly through MHC-II expression acting as antigen-presenting cells and causing chronic inflammation (7, 34).

Despite different cellular origin, STOSE and MOE tumors had high expression of MHC-II and PD-L1, which may be a particularity of the FVB/N genetic background as similar findings on MHC and PD-L1 expression were observed in bladder cancer (35). Comparison of ID8-WT and STOSE, two unmodified cell lines of similar origin, allowed us to directly assess the impact of mouse strain. There are inherent differences in the immune response reported in these strains, where C57BL/6 mice have a Th1 bias and FVB/N, like BALB/c, have a Th2 bias (36, 37). While both ID8-WT and STOSE derive from OSE, their tumors have distinct pathologies, with ID8-WT cells producing small primary tumors and large volumes of bloody ascites, whereas STOSE cells generate large primary tumors and viscous/mucous ascites. Furthermore, STOSE tumors are a unique, immunogenic, EOC model with a distinct TME that may be informative when testing the response to novel immunotherapies, especially those investigating TAMs.

Recent studies using syngeneic and genetically modified mouse models with homologous recombination (HR)-deficient DNA repair and P53 mutations have highlighted the heavy immune infiltration suggesting a more immunogenic phenotype (18, 38, 39), which is consistent with human disease (39, 40). However, neither ID8-p53^−/−^ nor the derivative cells with Brca2 deletion were immunogenic as determined by MHC expression and their inability to induce antitumoral protection when administered as a cellular vaccine. We observed more T-cell infiltration in ID8-p53^−/−^ tumors compared with ID8-WT tumors, but not compared with ID8-C3 tumors. Certainly, the tumor immune composition of ID8-WT and ID8-C3 models were often different, perhaps due to clonal selection and/or Cas9 expression, which can modulate innate/adaptive immune responses (41, 42). The similar immune composition in ID8-p53^−/−^ and ID8-C3 tumors, as reported by Walton and colleagues, indicates similar tumor development after intraperitoneal and intrabursal injection of cells (18). ID8-p53^−/−^ tumors were highly infiltrated by T cells, generally lacking expression of the activation markers CD25 and PD-1, but abundant in FOXP3, suggesting the presence of Tregs, which are associated with poor prognosis in human ovarian cancer (4). This model also recruited preferentially CD11b^+^CD11c^+^ cDC2s that can hamper the antitumoral responses by producing high amounts of IL10 (31).

Given that many HGSC tumors arise from the fallopian tube, we characterized the MOE-PTEN/KRAS tumors. Like STOSE, MOE-PTEN/KRAS tumors were highly infiltrated by myeloid cells, but these TAMs were primarily CD206 negative. Although both models derive from the FVB/N background, there were clear differences in the TME as a consequence of cell-intrinsic factors (cell type of origin/mutations). MOE-PTEN/KRAS tumors were highly infiltrated by CD4^+^ T cells, contained high frequencies of LAG3^+^ CD8^+^ T cells, and displayed significant expression of MHC-II. Double staining for MHC-II and cytokeratin 8/18 allowed the discrimination of cancer cells from MHC-II^+^ cells, suggesting other cellular sources of MHC-II expression in this model, such as adipocytes (34) or cancer associated fibroblasts (43). In colorectal cancer, *KRAS* mutation suppresses Th1/CTL immunity, reducing IFNγ and CXCL10 production, therefore decreasing CTL infiltration (44). MOE-PTEN/KRAS tumors displayed a shift toward Th2 CD4^+^ T cells with a high ratio of CD4/CD8 and produced very low amounts of CXCL10 in the ascites fluid. Overall, these tumors were poorly infiltrated by T cells, although we noted specific T-cell accumulation in clusters, potentially enabling T cell–activating responses. None of the other models had these T-cell clusters, suggesting an association with KRAS activation.

PD-L1 expression has been detected in 11%–60% of HGSC cases (45). In this study all orthotopic tumors expressed PD-L1, but in <3% of the tumor. ID8-p53^−/−^ and MOE-PTEN/KRAS models showed high presence and expression of PD-L1 among the immune cell populations while STOSE showed high PD-L1 expression in nonimmune cells. Given low response rates to ICIs for the treatment of ovarian cancer (9, 10), testing ICIs targeting the PD-1/PD-L1 blockade, alone or in combination with other strategies, in these models may help to stratify those women who may respond to treatment. Studies investigating genetic alterations and response to ICIs have shown that PTEN loss results in resistance to PD-1 blockade in melanoma and uterine leiomyosarcoma (46, 47). In lung adenocarcinoma, *TP53* and *KRAS* mutations are key factors affecting PD-L1 expression and sensitivity to PD-1 blockade (48). Future experiments treating a broad range of existing murine EOC models (19, 22, 38, 39) with ICI therapy could better elucidate the specific role that these mutations play in response to immunotherapy.

In this study, we characterized the immune cell populations, cytokine expression, MHC expression, and immunogenicity of several syngeneic models, demonstrating that they possess unique TMEs. ID8-C3 and ID8-p53^−/−^ models were more T-cell infiltrated compared with the ID8-WT and STOSE, while STOSE and MOE-PTEN/KRAS tumors were heavily infiltrated by TAMs. Based on our findings, immunotherapeutic approaches can be proposed to determine their efficacy in association with the immune characteristics of each model (Supplementary Table S9). For example, differences in MHC-I and -II, as well as PD-L1 may suggest which models will respond to ICIs. This comparative analysis provides a solid foundation to understand the shared and distinct features of a set of syngeneic models that have significant potential for future testing of novel immunotherapies for human patients with similar profiles in their TME.

## Supplementary Material

Supplementary Figures S1-S13 Figure S1 shows differential gene expression between cancer cell populations of orthotopic ID8-WT and STOSE tumors.
Figure S2 shows UMAP plots of M0/M1/M2 gene set enrichment in ID8-WT and STOSE tumors.
Figure S3 shows expression of MHC-I and PD-L1 under IFNγ treatment in various ovarian cancer cell lines.
Figure S4 shows tumor phenotypes of various murine ovarian cancer models.
Figure S5 shows the gating strategy for analysis of flow cytometry data.
Figure S6 shows abundance of T cell populations in the TME of syngeneic ovarian cancer models.
Figure S7 shows abundance of myeloid-like cell populations in the TME of various syngeneic ovarian cancer models.
Figure S8 shows MHC-II expression in immune and stromal compartments of orthotopic tumors from syngeneic ovarian cancer models.
Figure S9 shows PD-L1 expression in immune and stromal compartments of orthotopic tumors from syngeneic ovarian cancer models.
Figure S10 shows relative immune cell frequencies found in spleens of ovarian tumor-bearing mice.
Figure S11 shows MHC-II and PD-L1 expression in the TME and ascites of ovarian tumor-bearing mice.
Figure S12 shows the chemokine and cytokine network found in the ascites and plasma of ovarian tumor-bearing mice.
Figure S13 shows chemokine and cytokine expression from single-cell RNA-sequencing analysis of ID8 and STOSE tumors.

Supplementary Tables S1-S9 Table S1 contains antibodies used in flow cytometry staining of ascites/PW, spleen, and mouse ovarian tumors.
Table S2 contains primary antibodies used for IHC and IF staining of mouse ovarian tumors.
Table S3 contains primer Sequences used for qPCR of ovarian cancer cell lines.
Table S4 contains hallmark gene list for ID8 and STOSE cancer cells.
Table S5 contains M0/M1/M2 gene-sets.
Table S6 contains tumor development characteristics of murine orthotopic ovarian cancer models.
Table S7 contains average and standard deviation of concentrations of cytokines and chemokines in the ascites of syngeneic mouse models of ovarian cancer at endpoint.
Table S8 contains average and standard deviation of concentrations of cytokines and chemokines in the plasma of syngeneic mouse models of ovarian cancer collected near endpoint.
Table S9 summarizes key features of syngeneic models of ovarian cancer.
