# Supplementary material for: The Tumor Immune Profile of Murine Ovarian Cancer Models: An Essential Tool for Ovarian Cancer Immunotherapy Research
Source: Cancer Res Commun. 2022 Jun 9;2(6):417–33. doi: 10.1158/2767-9764.CRC-22-0017 (PMC9616009; doi:10.1158/2767-9764.CRC-22-0017)
Supplement: Supplementary Figures S1-S13 — Figure S1 shows differential gene expression between cancer cell populations of orthotopic ID8-WT and STOSE tumors. Figure S2 shows UMAP plots of M0/M1/M2 gene set enrichment in ID8-WT and STOSE tumors. Figure S3 shows expression of MHC-I and PD-L1 under IFNγ treatment in various ovarian cancer cell lines. Figure S4 shows tumor phenotypes of various murine ovarian cancer models. Figure S5 shows the gating strategy for analysis of flow cytometry data. Figure S6 shows abundance of T cell populations in the TME of syngeneic ovarian cancer models. Figure S7 shows abundance of myeloid-like cell populations in the TME of various syngeneic ovarian cancer models. Figure S8 shows MHC-II expression in immune and stromal compartments of orthotopic tumors from syngeneic ovarian cancer models. Figure S9 shows PD-L1 expression in immune and stromal compartments of orthotopic tumors from syngeneic ovarian cancer models. Figure S10 shows relative immune cell frequencies found in spleens of ovarian tumor-bearing mice. Figure S11 shows MHC-II and PD-L1 expression in the TME and ascites of ovarian tumor-bearing mice. Figure S12 shows the chemokine and cytokine network found in the ascites and plasma of ovarian tumor-bearing mice. Figure S13 shows chemokine and cytokine expression from single-cell RNA-sequencing analysis of ID8 and STOSE tumors. [file crc-22-0017-s01.pdf]

SUPPLEMENTARY FIGURES

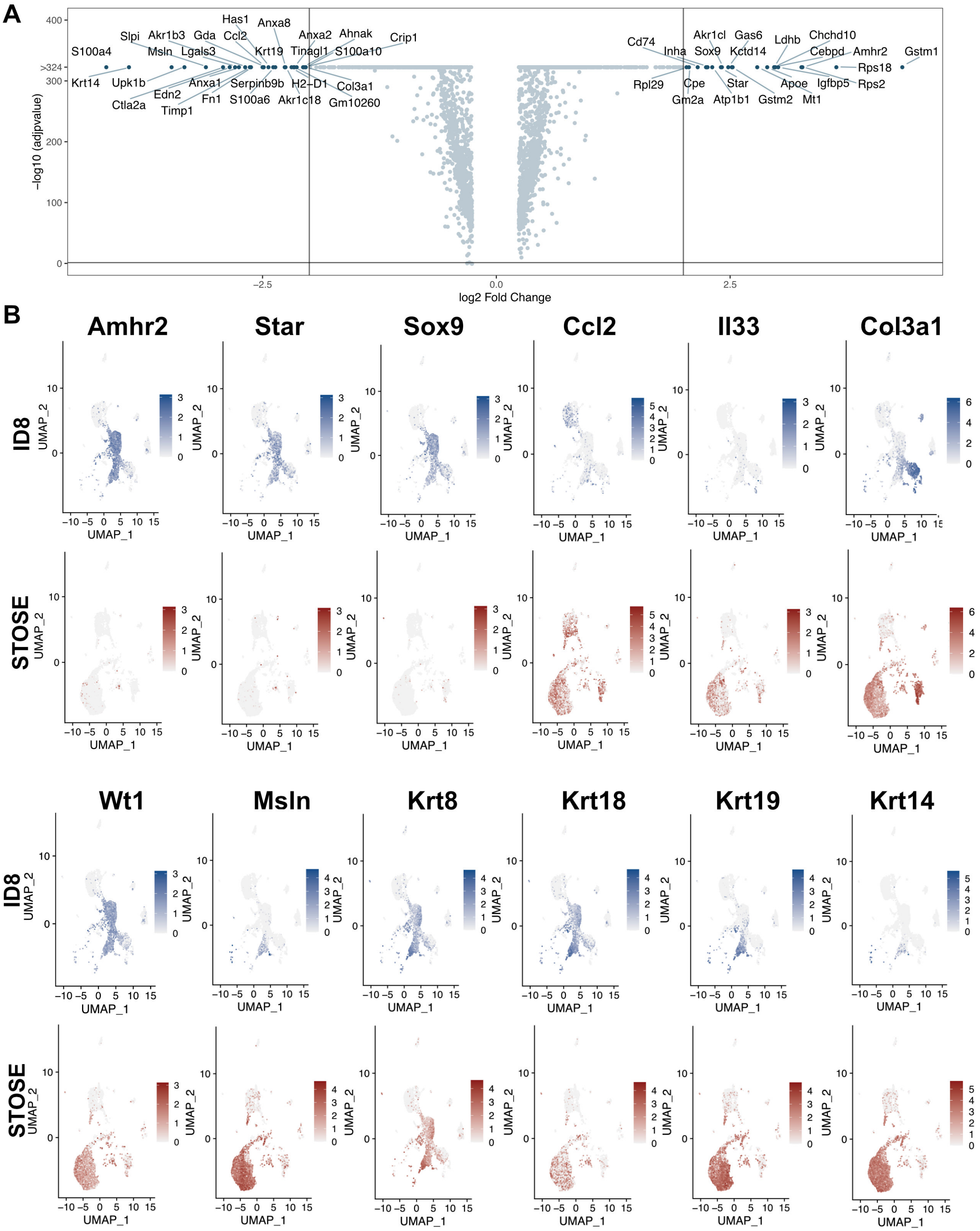

Figure S1: Differential gene expression in ID8 and STOSE tumors. (A) Volcano plot showing the most differentially expressed genes (DGEs) between cancer cell populations of orthotopic ID8-WT (left) and STOSE-WT (right) tumors beyond log2 fold-change threshold of 2. DGEs calculated using MAST test comparing ID8 and STOSE cancer cells. P-values were assigned a lower bound of p=1e-324. (B) UMAPs showing expression of *Amhr2, Star, Sox9, Il33, Ccl2, Col1a3, Wt1, Msln, Krt8, Krt18, Krt19, Krt14* in ID8-WT (upper panels) and STOSE (lower panels) orthotopic tumors. Heatmap displays the level of expression in cell types in ID8-WT (blue) and STOSE (red) samples.

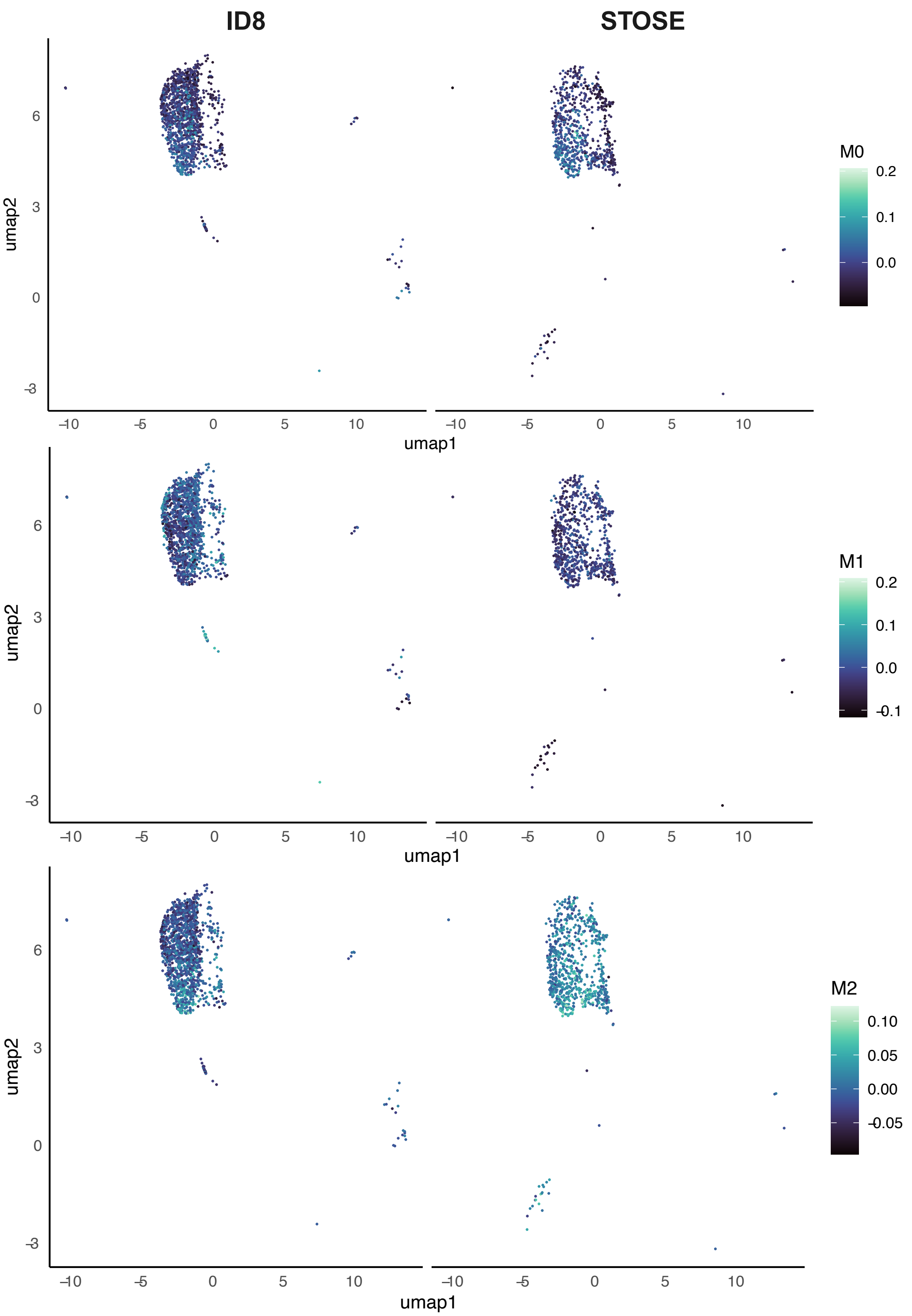

Figure S2: M0/M1/M2 gene set enrichment in ID8 and STOSE tumors. UMAP plot showing enrichment of macrophages for gene-sets (Table S5) generated for M0, M1, M2 polarization.

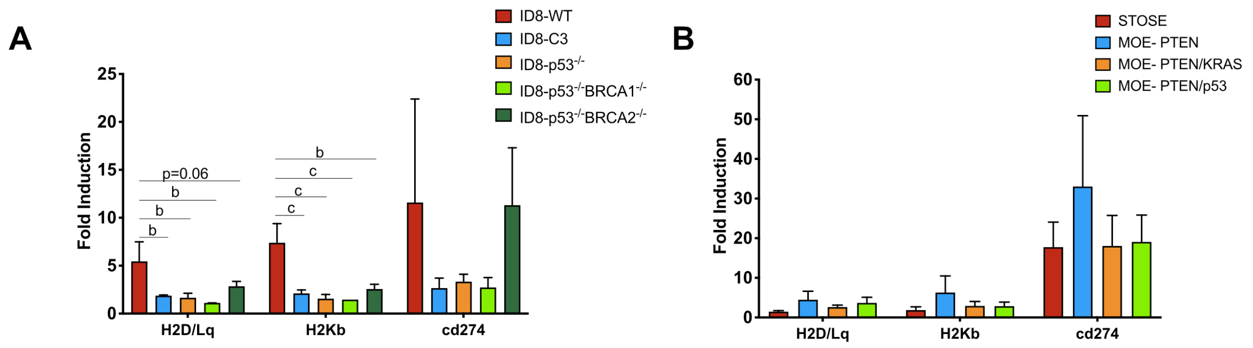

Figure S3: Expression of MHC-I and PD-L1 in various ovarian cancer cell lines. H2K, H2D, and CD274 expression under IFNγ treatment for (A) C57BL/6 and (B) FVB/N-derived cell lines. Fold induction was determined by RT-qPCR for each cell line relative to untreated samples and normalized to the housekeeping gene *Rplp0*. Cells were treated with 500 pg/mL IFNγ for 24 hours. n=3 independent replicates. Significance was determined by one-way ANOVA with Tukey's post-test, b: $p<0.01$; c: $p<0.001$.

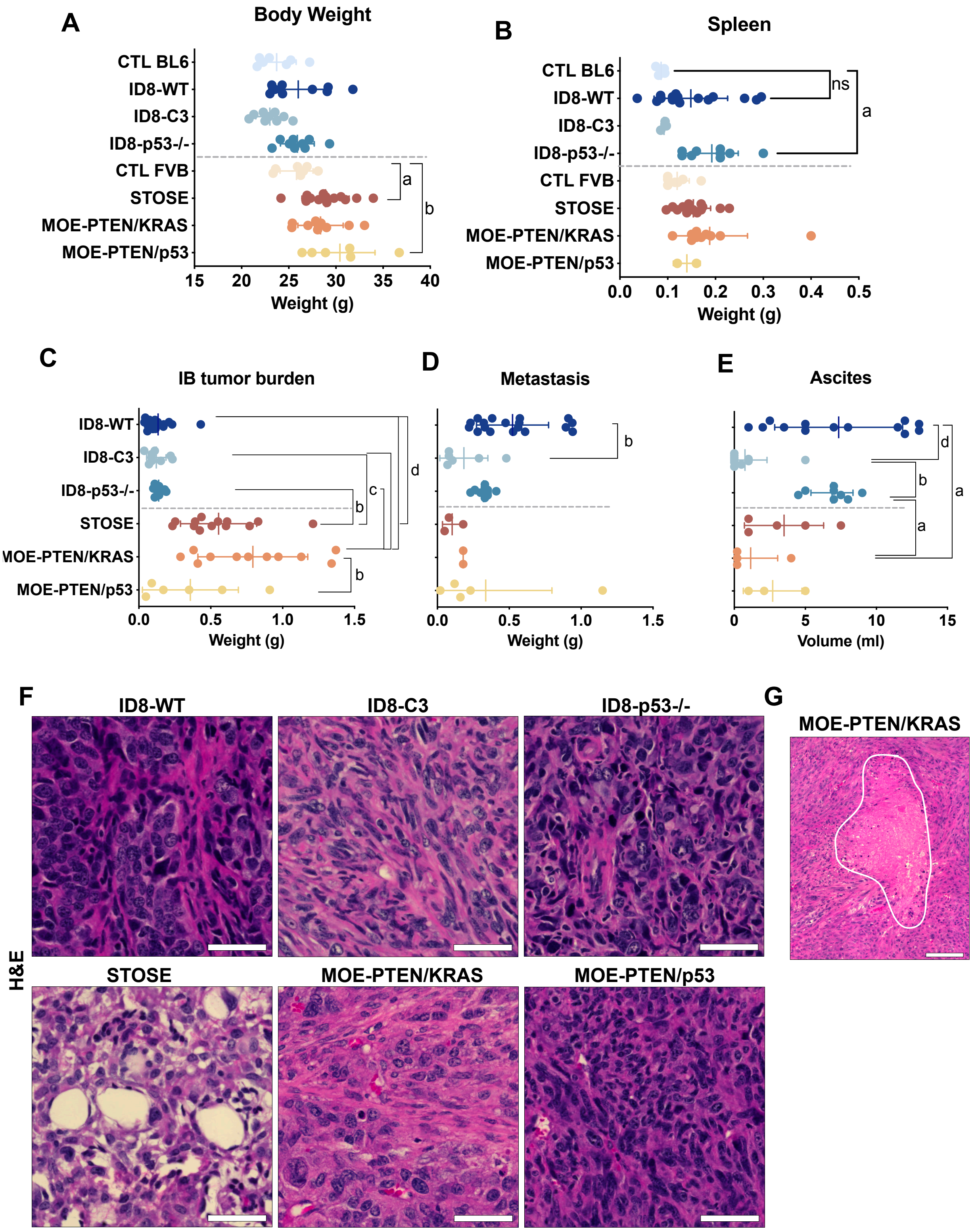
A
Body Weight
CTL BL6
ID8-WT
ID8-C3
ID8-p53-/-
CTL FVB
STOSE
MOE-PTEN/KRAS
MOE-PTEN/p53
15 20 25 30 35 40
Weight (g)
a
b
B
Spleen
ns
a
0.0 0.1 0.2 0.3 0.4 0.5
Weight (g)
C
IB tumor burden
d
c
b
0.0 0.5 1.0 1.5
Weight (g)
D
Metastasis
Weight (g)
E
Ascites
0 5 10 15
Volume (ml)
F
H&E
G

Figure S4: Tumor phenotype of various murine ovarian cancer models. Mice were euthanized 4-5 days before reaching humane endpoint and the mass of (A) mice and (B) spleen weights, (C) primary tumors, and (D) metastases, as well as (E) ascites volume were collected and recorded. Each dot represents an individual sample. Mean values with SD are shown for each tumor model. Statistical significance was determined using one-way ANOVA with Tukey's multiple comparison test, a: $p<0.05$; b: $p<0.01$; and c: $p<0.001$. (F) Representative histological sections of hematoxylin and eosin staining displaying overall characteristics of the tumor niche of each orthotopic tumor. Images are representative of ID8-WT (n=9), ID8-C3 (n=6), ID8-p53-/- (n=9), STOSE (n=12), MOE-PTEN/KRAS (n=6), MOE-PTEN/p53 (n=6) tumors. Scale bars = 50µm. (G) Representative image of MOE-PTEN/KRAS tumor with hematoxylin and eosin staining with a region of necrosis outlined. Scale bars = 200µm.

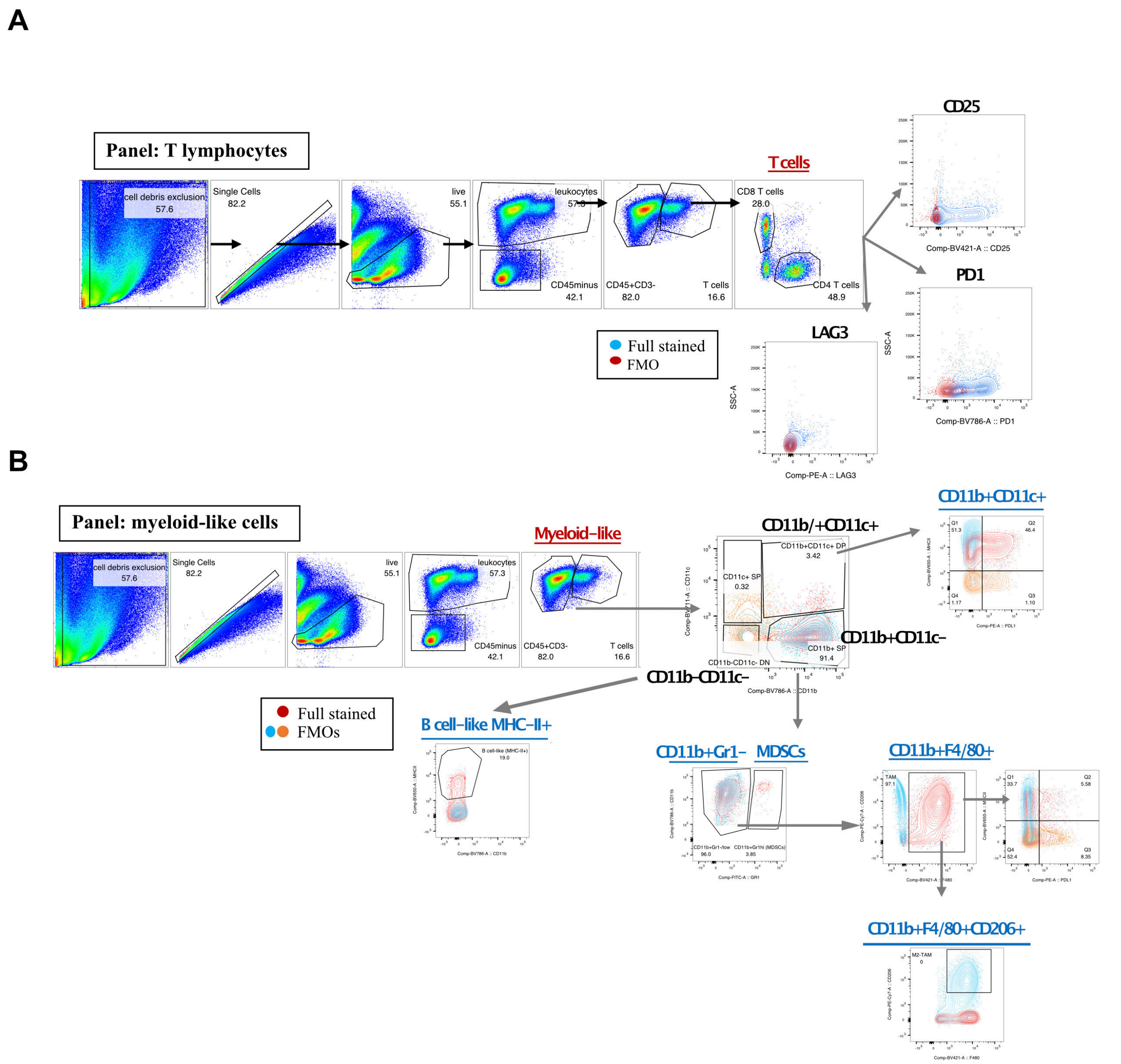

Figure S5: Gating strategy for analysis of flow cytometry data. Orthotopic murine tumors, spleens and peritoneal fluid were collected near the endpoint and processed to be analyzed by flow cytometry or IHC. (A) Gating strategy used to analyze the flow cytometry data as follows: cell debris exclusion, singlet, live/dead exclusion, leukocytes (CD45+), CD3- (myeloid-like), CD3+ (T cells). Other markers included in the T-cell panel were CD4, CD8, PD1, LAG3 and CD25 (see Table S1). (B) In the "myeloid-like" panel, the following markers were used to further identify other immune subsets: CD11b, CD11c, Gr1, MHC-II-, PD-L1, F4/80, and CD206 (see Table S1). CD11c+ includes lymphoid-lineage DCs; CD11b+CD11c+ myeloid-lineage DCs. The subset CD11b+CD11c- was further gated for Gr1 high/low expression to discriminate between MDSCs (CD11b+Gr1+) and CD11b+Gr1-/low cell types (monocytes, macrophages). Further gating in the CD11b+Gr1- subpopulation allowed for the identification of TAMs (CD11b+F4/80+) and CD206+ TAMs (M2-like macrophages) within the same population. Contour plots representative of fluorescence minus one (FMOs) for each analyzed marker.

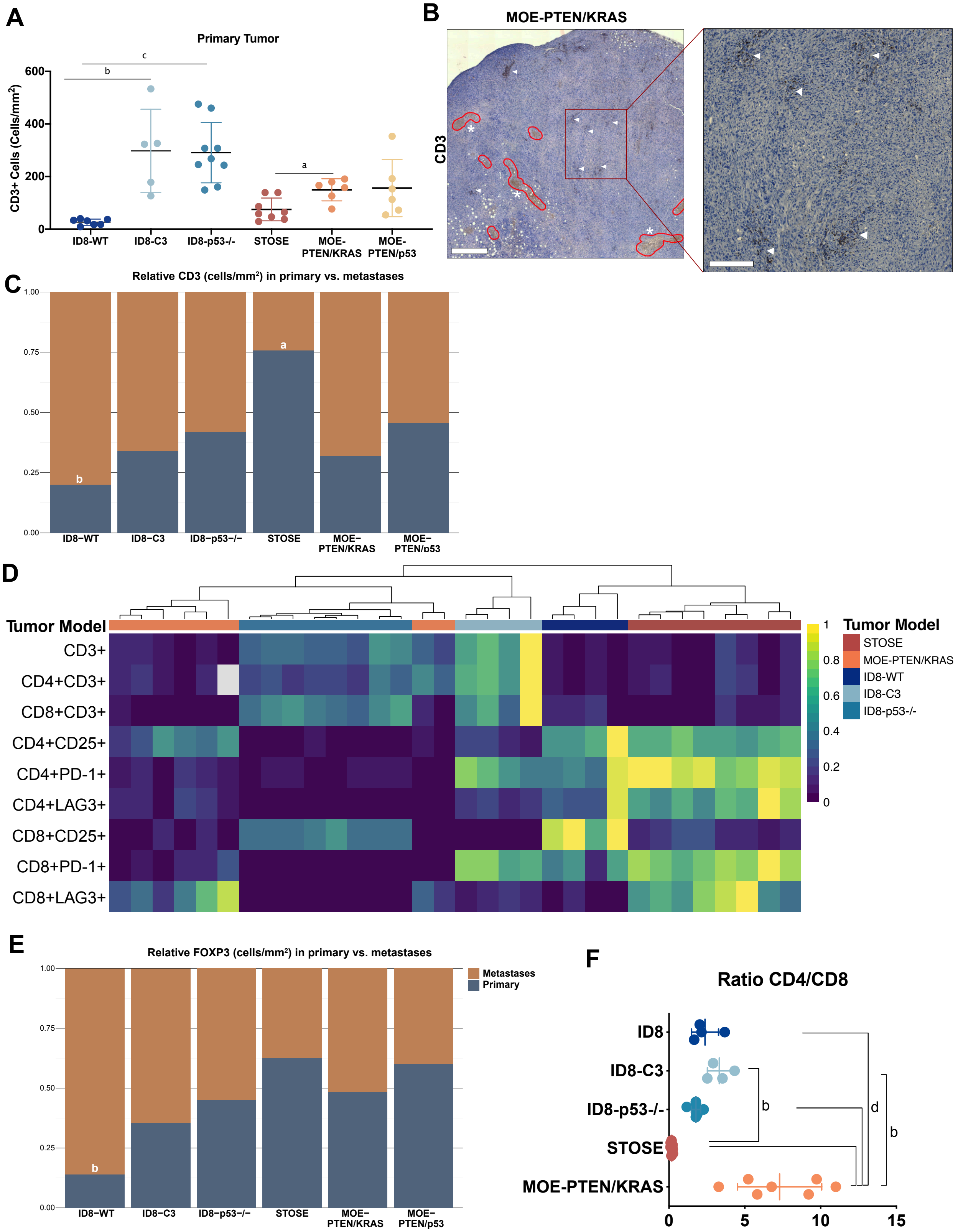
A
Primary Tumor
CD3+ Cells (Cells/mm²)
600
400
200
0
c
b
a
ID8-WT
ID8-C3
ID8-p53-/-
STOSE
MOE-PTEN/KRAS
MOE-PTEN/p53
B
MOE-PTEN/KRAS
CD3
C
Relative CD3 (cells/mm²) in primary vs. metastases
1.00
0.75
0.50
0.25
0.00
a
b
ID8−WT
ID8−C3
ID8−p53−/−
STOSE
MOE−PTEN/KRAS
MOE−PTEN/p53
D
Tumor Model
CD3+
CD4+CD3+
CD8+CD3+
CD4+CD25+
CD4+PD-1+
CD4+LAG3+
CD8+CD25+
CD8+PD-1+
CD8+LAG3+
1
0.8
0.6
0.4
0.2
0
Tumor Model
STOSE
MOE-PTEN/KRAS
ID8-WT
ID8-C3
ID8-p53-/-
E
Relative FOXP3 (cells/mm²) in primary vs. metastases
Metastases
Primary
1.00
0.75
0.50
0.25
0.00
b
ID8−WT
ID8−C3
ID8−p53−/−
STOSE
MOE−PTEN/KRAS
MOE−PTEN/p53
F
Ratio CD4/CD8
ID8
ID8-C3
ID8-p53-/-
STOSE
MOE-PTEN/KRAS
b
d
b
0
5
10
15

Figure S6: Abundance of T cells in the TME of syngeneic ovarian cancer models. (A) Quantification of CD3+ cells by IHC. Cell counts (number of cells/mm^2) were quantified using ImagePro Premier. Each dot represents a single tumor sample as follows: ID8-WT (n=8), ID8-C3 (n=6), ID8-p53-/- (n=9), STOSE (n=8), MOE-PTEN/KRAS (n=6), MOE-PTEN/p53 (n=6). Mean values with SD are shown for each tumor model. Significance was determined by one-way ANOVA within C57BL/6 or FVB/N models with Tukey's post-test or a two-tailed Student's t-test (comparing ID8 and STOSE), a: $p<0.05$; b: $p<0.01$; c: $p<0.001$. (B) Representative images of IHC for CD3 staining of MOE-PTEN/KRAS tumor sample revealing CD3 clusters. Scale bars = 1mm (left panel) and 200µm (right panel). White arrowheads in left panel indicating clusters of CD3+ cells are further magnified in the right panel. Asterisks (*) indicate outlined necrotic areas deprived of cells with non-specific staining. (C) Relative abundance of CD3+ cells in primary vs. metastatic tumors. Metastatic tumors were stained and quantified as with primary tumors. ID8-WT (n=9), ID8-C3 (n=6), ID8-p53-/- (n=10), STOSE (n=6), MOE-PTEN/KRAS (n=3), MOE-PTEN/p53 (n=5). Significance was determined by Student's t-test, a<0.05, b<0.01. (D)Heatmap depicting normalized relative frequencies of T cell subsets as determined by flow cytometry for all tumor models. White square is an omitted outlier sample. (E) Relative abundance of FOXP3+ cells in primary vs. metastatic tumors. Metastatic tumors were stained and quantified as with primary tumors presented in 2E. Data representative of ID8-WT (n=10), ID8-C3 (n=5), ID8-p53-/- (n=9), STOSE (n=7), MOE-PTEN/KRAS (n=3), MOE-PTEN/p53 (n=5) samples. Significance was determined by Student's t-test, b<0.01. (F) Ratio of CD4/CD8 T cells. Each dot represents a single sample derived from the supernatant of ascites from tumor-bearing mice. Mean values with SD are shown for each tumor model. Significance was determined by one-way ANOVA with Tukey's post-test comparing all models, b: $p<0.01$; d: $p<0.0001$.

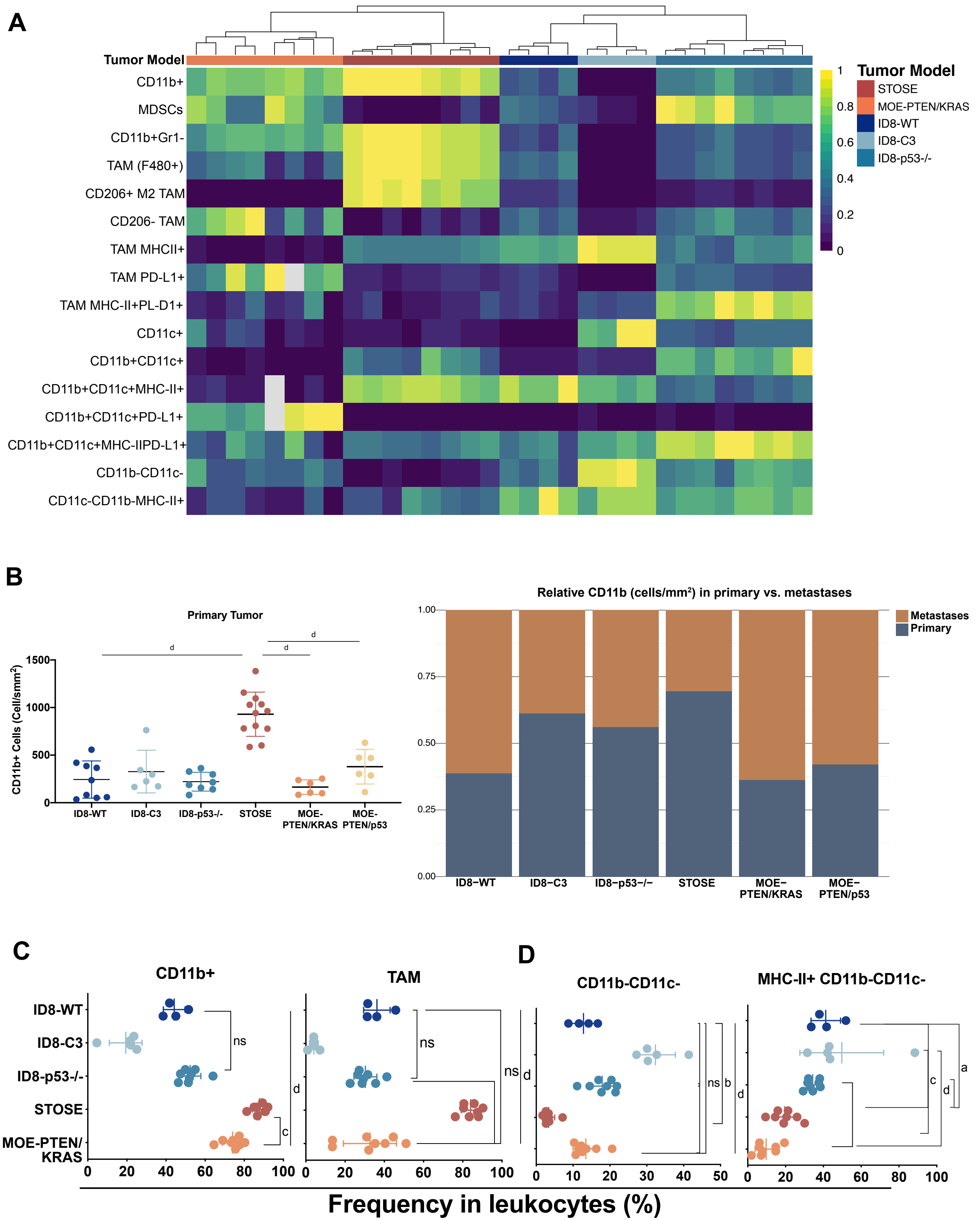

A
Tumor Model
CD11b+
MDSCs
CD11b+Gr1-
TAM (F480+)
CD206+ M2 TAM
CD206- TAM
TAM MHCII+
TAM PD-L1+
TAM MHC-II+PL-D1+
CD11c+
CD11b+CD11c+
CD11b+CD11c+MHC-II+
CD11b+CD11c+PD-L1+
CD11b+CD11c+MHC-IIPD-L1+
CD11b-CD11c-
CD11c-CD11b-MHC-II+
1
0.8
0.6
0.4
0.2
0
Tumor Model
STOSE
MOE-PTEN/KRAS
ID8-WT
ID8-C3
ID8-p53-/-
B
Primary Tumor
CD11b+ Cells (Cell/smm2)
1500
1000
500
0
d
d
d
ID8-WT
ID8-C3
ID8-p53-/-
STOSE
MOE-PTEN/KRAS
MOE-PTEN/p53
Relative CD11b (cells/mm²) in primary vs. metastases
1.00
0.75
0.50
0.25
0.00
Metastases
Primary
ID8−WT
ID8−C3
ID8−p53−/−
STOSE
MOE−PTEN/KRAS
MOE−PTEN/p53
C
CD11b+
TAM
ID8-WT
ID8-C3
ID8-p53-/-
STOSE
MOE-PTEN/KRAS
ns
d
c
ns
ns
d
0 20 40 60 80 100
0 20 40 60 80 100
D
CD11b-CD11c-
MHC-II+ CD11b-CD11c-
ns
b
d
c
d
a
0 10 20 30 40 50
0 20 40 60 80 100
Frequency in leukocytes (%)

Figure S7: Abundance of myeloid-like cells in the TME of various syngeneic ovarian cancer models. (A) Heatmap depicting normalized relative frequency of the myeloid-like compartment for all tumor models, determined by flow cytometry. White squares are omitted outlier samples. (B) Cell counts (number of cells/mm^2) were quantified using ImagePro Premier. Each dot represents a single tumor sample as follows: ID8-WT (n=9), ID8-C3 (n=6), ID8-p53-/- (n=8), STOSE (n=12), MOE-PTEN/KRAS(n=6), and MOE-PTEN/p53 (n=6). Relative abundance of CD11b+ cells in primary vs. metastatic tumors as determined by immunohistochemistry. Metastatic tumors were stained and quantified as with primary tumors. ID8-WT (n=10), ID8-C3 (n=5), ID8-p53-/- (n=9), STOSE (n=6), MOE-PTEN/KRAS (n=3), MOE-PTEN/p53 (n=5). No significant difference was found by Student's t-test. (C-E) Total frequencies in leukocytes of total (C) F480+ TAMs and (D) CD11b-CD11c- cells with a MHC-II+ fraction. Each dot represents an orthotopic tumor. Mean values with SD are shown. Significance was determined by one-way ANOVA with Tukey's post-test comparing all models, a: $p<0.05$, b: $p<0.01$; c: $p<0.001$; d: $p<0.0001$.

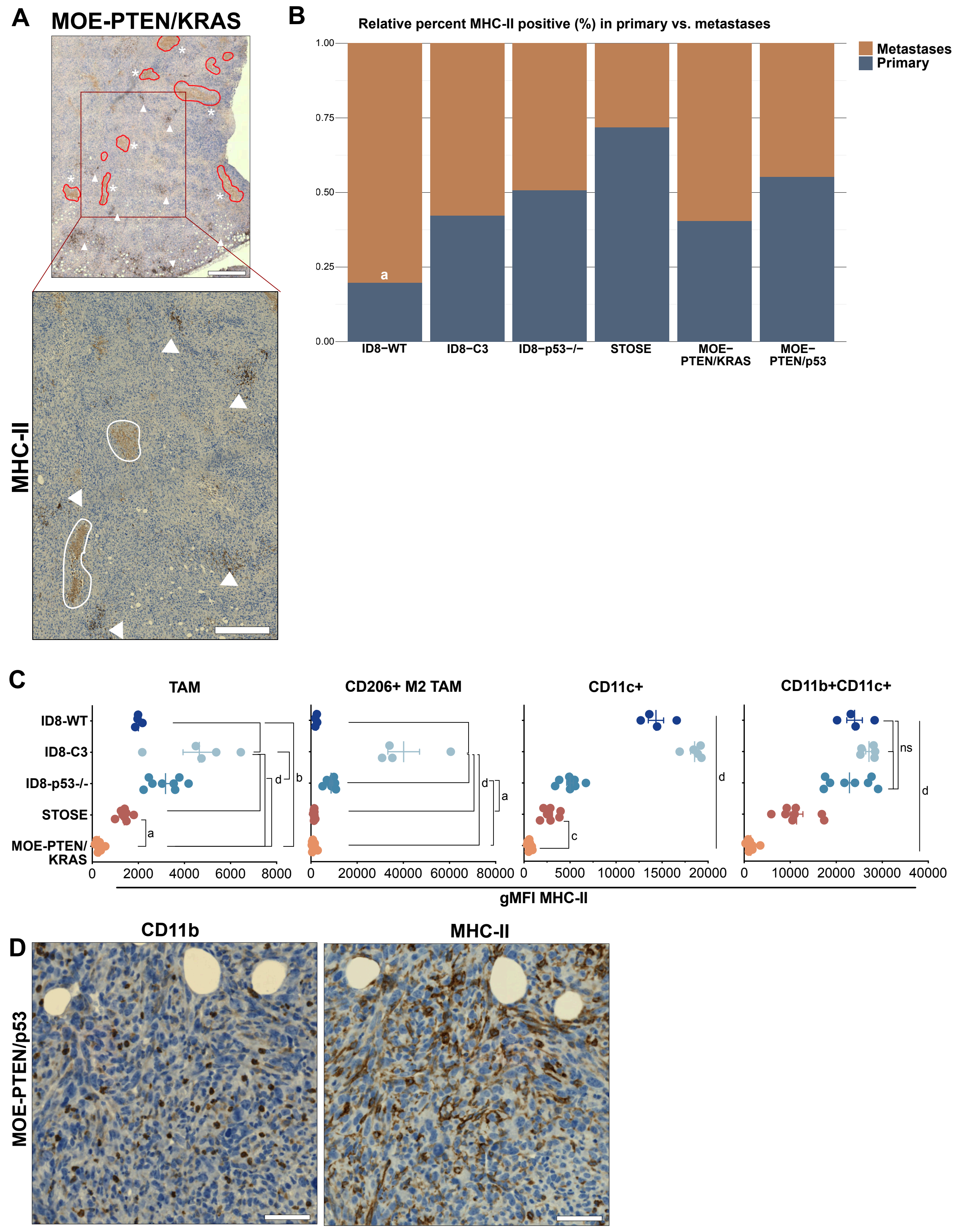
A
MOE-PTEN/KRAS
MHC-II
B
Relative percent MHC-II positive (%) in primary vs. metastases
Metastases
Primary
1.00
0.75
0.50
0.25
0.00
a
ID8−WT
ID8−C3
ID8−p53−/−
STOSE
MOE−PTEN/KRAS
MOE−PTEN/p53
C
TAM
CD206+ M2 TAM
CD11c+
CD11b+CD11c+
ID8-WT
ID8-C3
ID8-p53-/-
STOSE
MOE-PTEN/KRAS
a
b
c
d
ns
0
2000
4000
6000
8000
20000
40000
60000
80000
5000
10000
15000
20000
10000
20000
30000
40000
gMFI MHC-II
D
CD11b
MHC-II
MOE-PTEN/p53

Figure S8: MHC-II expression in immune and stromal compartments of orthotopic tumors from syngeneic ovarian cancer models. (A) Representative images of IHC for MHC-II staining of MOE-PTEN/KRAS tumors revealing MHC-II+ clusters. Scale bars = 1mm (top panel) and 200µm (bottom panel). White arrowheads in the top panel indicating clusters of MHC-II+ cells are further magnified in the bottom panel. Asterisks (*) indicate outlined necrotic areas deprived of cells with non-specific staining. Sections were counterstained with hematoxylin (blue) and positive cells (brown) were stained with DAB. Scale bars = 50µm. (B) Relative abundance of MHC-II as detected by IHC in primary vs. metastatic tumors. Metastatic tumors were stained and quantified as with primary tumors presented in 6A. ID8-WT (n=10), ID8-C3 (n=5), ID8-p53-/- (n=9), STOSE (n=7), MOE-PTEN/KRAS (n=3), MOE-PTEN/p53 (n=5). Significance was determined by Student's t-test, a<0.05. (C) Expression of MHC-II+ in TAMs, CD206+ M2 CD206+ TAMS, CD11c+ and CD11b+CD11c+ cells in primary tumors as assessed by flow cytometry. Mean values with SEM are shown. Significance was determined by one-way ANOVA with Tukey's post-test comparing all models, a: p<0.05, b: p<0.01; c: p<0.001; d: p<0.0001.(D) Representative IHC images of CD11b and MHC-II staining in MOE-PTEN/p53 tumors show MHC-II expression by CD11b negative cells. Images are from serial sections of the same tumor. Images representative of n=6 primary and n=5 metastatic MOE-PTEN/p53 tumors.

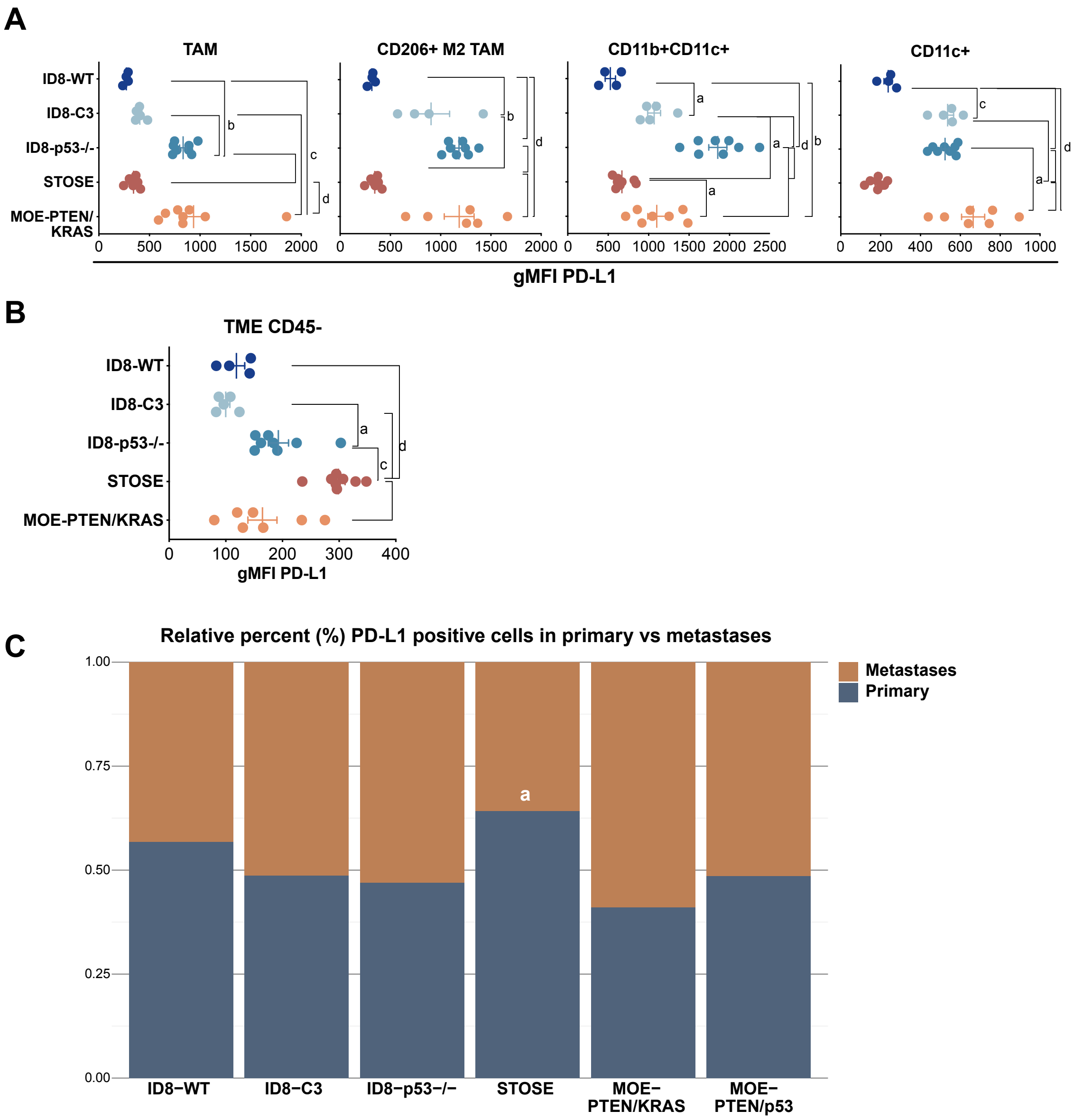

Figure S9: PD-L1 expression in immune and stromal compartments of orthotopic tumors from syngeneic ovarian cancer models. (A) PD-L1 expression assessed by gMFI by flow cytometry in TAMs, M2 CD206+ TAMs, CD11b+CD11c+, and CD11c+ immune subsets found in the TME. Each dot represents a single sample derived from an orthotopic tumor-bearing or control mouse. Cells were analyzed as shown in Figure S5B. (B) gMFI of PD-L1 expression on CD45-negative population in the TME. Mean values with SD (A) or SEM (B) are shown for each tumor model. Significance was determined by one-way ANOVA with Tukey's post-test comparing all models, a: p<0.05, b: p<0.01; c: p<0.001; d: p<0.0001. (C) Relative abundance of PD-L1 expression as detected by IHC in primary vs. metastatic tumors. Metastatic tumors were stained and quantified as with primary tumors presented in Figure 6D. Data representative of ID8-WT (n=9), ID8-C3 (n=6), ID8-p53-/- (n=9), STOSE (n=11), MOE-PTEN/KRAS (n=3), MOE-PTEN/p53 (n=5) samples. Significance was determined by Student's t-test, a<0.05.

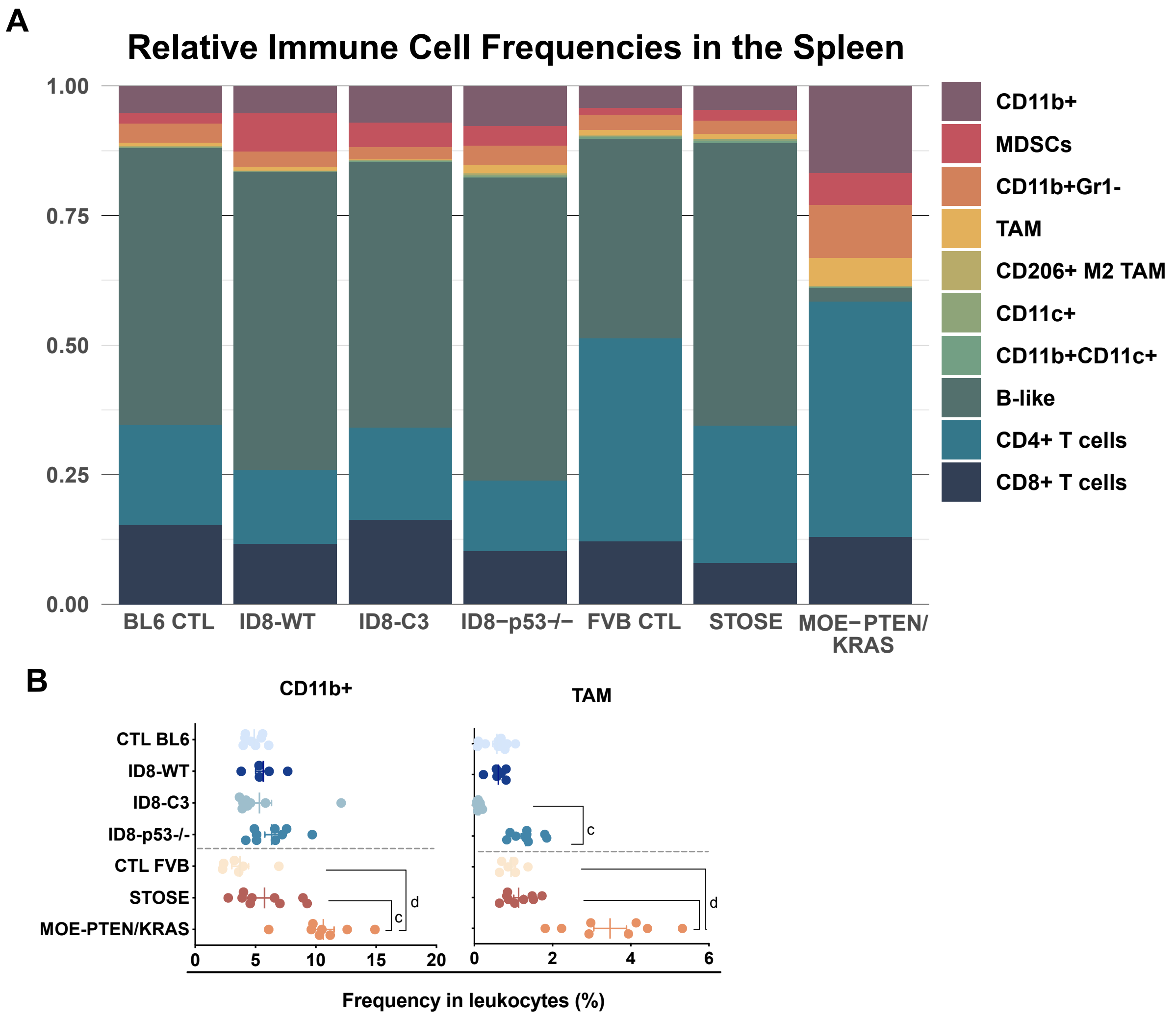

Figure S10: Relative immune cell frequencies found in spleens of ovarian tumor-bearing mice. (A) Stacked-bars figures showing the relative frequencies of several immune populations found in the spleens of tumor-bearing mice, near the endpoint, as assessed by flow cytometry (see Figure S5). Samples from age-matched tumor-naive mice were added as controls for each mouse strain. (B) Frequency of CD11b+ cells and TAMs in the spleens of all models. Each dot represents a single sample derived from an orthotopic tumor-bearing or control mouse. Mean values with SEM are shown. Significance was determined by one-way ANOVA with Tukey's post-test comparing all models, a<0.05, b<0.01. a: p<0.05, b: p<0.01; c: p<0.001.

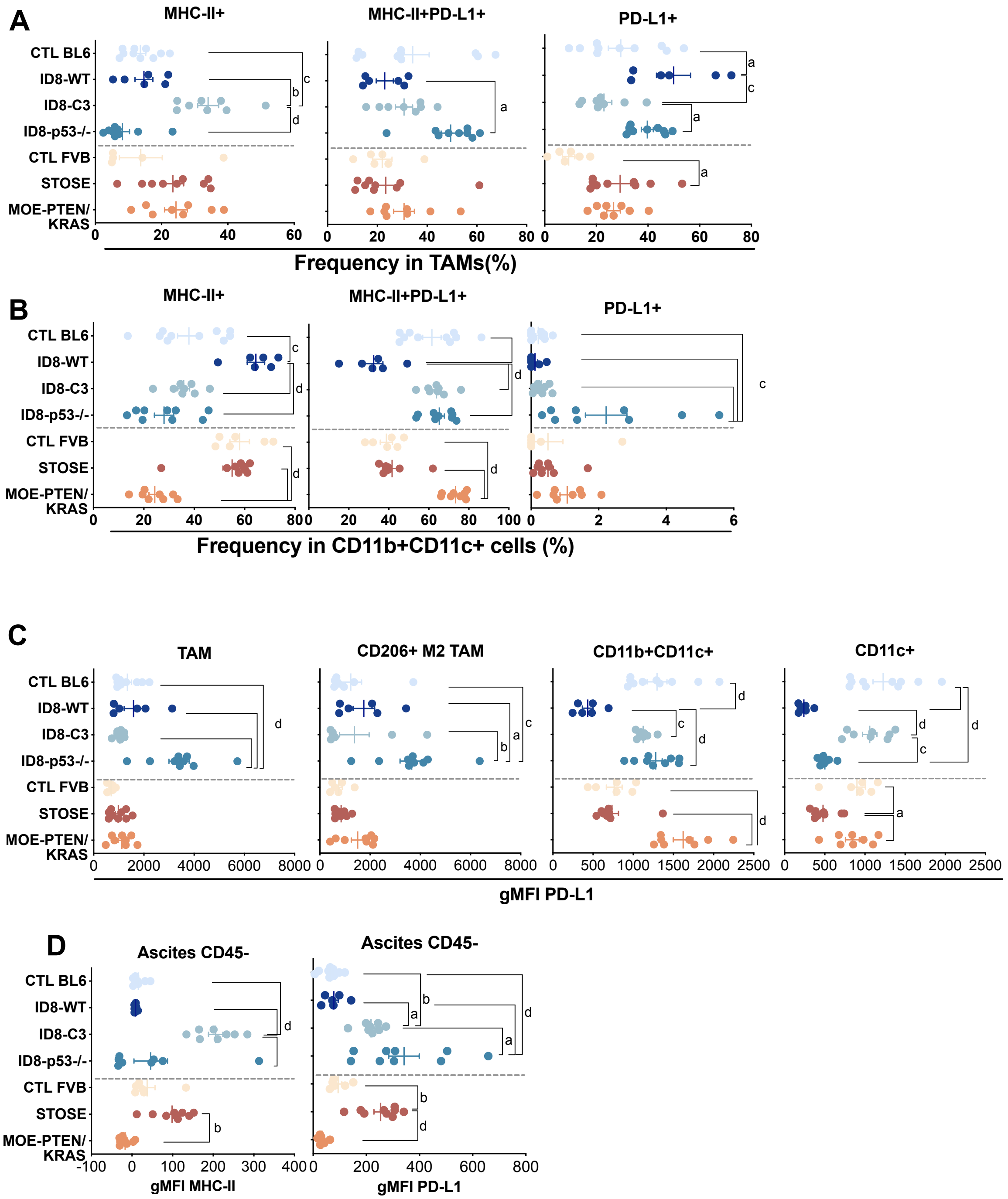

Figure S11: MHC-II and PD-L1 expression in the TME and ascites of ovarian tumor-bearing mice. For the ascites analysis, peritoneal derived fluid from age-matched tumor-naive mice were included as controls for each mouse strain. Each dot represents a single sample derived from an orthotopic tumor-bearing or control mouse. Cells were analyzed as shown in Figure S5 and for each subpopulation, MHC-II or PD-L1 frequency was assessed. Frequency of MHC-II+, MHC-II+PD-L1+, and PD-L1 (A) TAMs and (B) CD11b+CD11c+ subsets in the ascites fluid. (C) PD-L1 expression assessed by gMFI by flow cytometry in TAMs, M2 CD206+ TAMs,

CD11b+CD11c+, and CD11c+ immune subsets in the ascites. Mean values with SEM are shown. Significance was determined by one-way ANOVA with Tukey's post-test comparing all models, a: $p<0.05$, b: $p<0.01$; c: $p<0.001$; d: $p<0.0001$. (D) gMFI of PD-L1 expression on CD45-negative population in ascites.

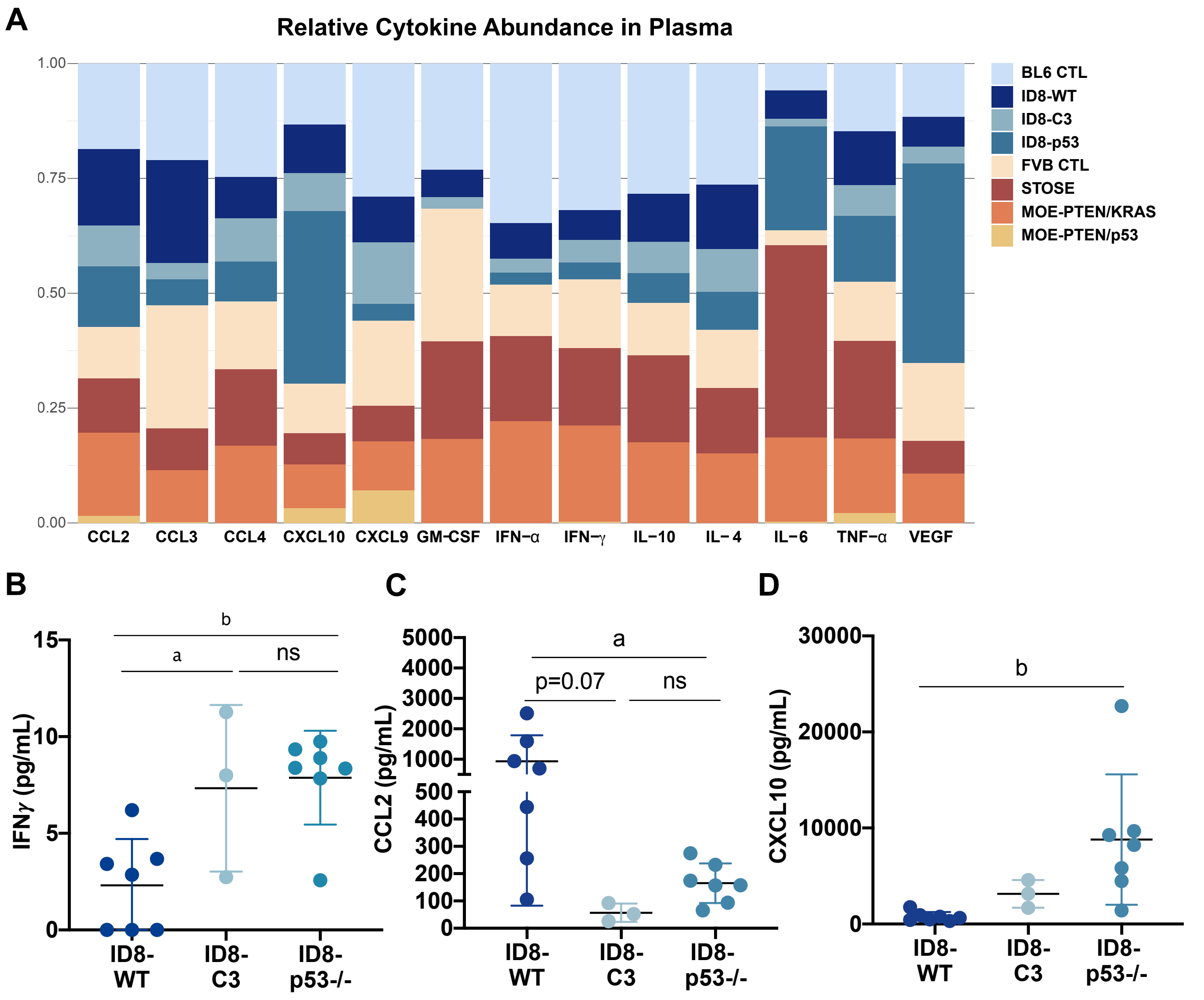

Figure S12: Chemo/cytokine network found in the ascites and plasma of ovarian tumor-bearing mice. (A) Stacked-bars figure showing the relative abundance of cytokines and chemokines in the plasma derived from orthotopic tumor-bearing mice. (B) IFNγ, (C) CCL2, and (D) CXCL10 in the ascites fluid (pg/ml) of ID8-derived models. Chemo/cytokines were measured by LEGENDplex™ Mouse Cytokine Release Syndrome Panel (13-plex) Multi-Analyte Flow Assay. Each dot represents a single sample derived from ascites fluid (B-D) from tumor-bearing mice. Mean values with SD are shown for each tumor model. Significance was determined by one-way ANOVA with Tukey's post-test, ns: not significant, a: $p<0.05$, b: $p<0.01$.

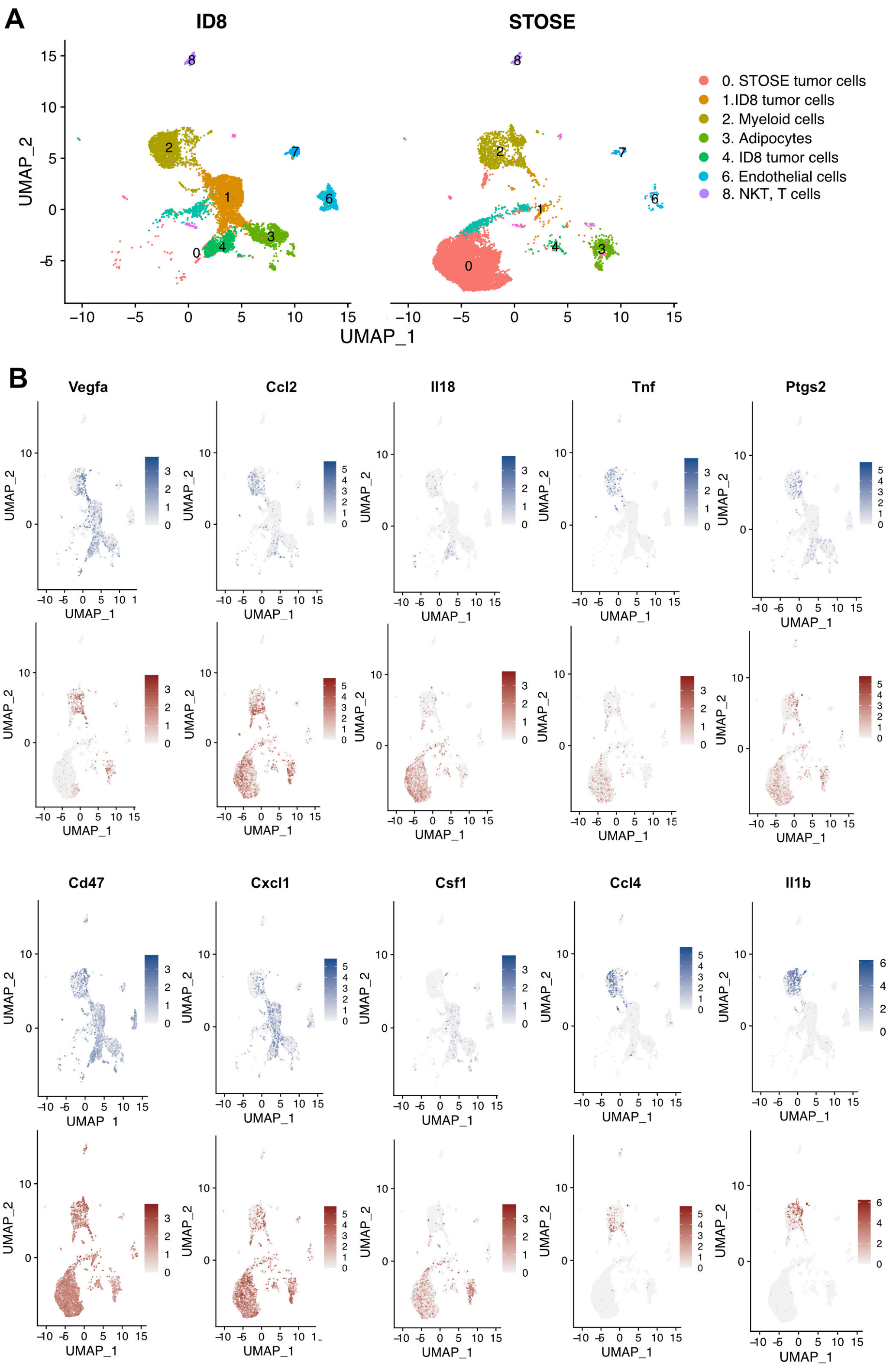

Figure S13: Single-cell RNA-sequencing analysis of cytokines/chemokine expression in ID8 and STOSE tumors. (A) Single cell RNA-sequencing UMAP figures depicting cell clusters found

in orthotopic ID8-WT (left) and STOSE (right) tumors at endpoint. (B) UMAPs showing expression of *Vegfa, Ccl2, Ccl4, Tnf, Ptgs2, Il18, Il1b, CxCl1, Csf1*, and *Cd47* in ID8-WT (upper panels) and STOSE (lower panels) orthotopic tumors. Heatmap displays the level of expression in cell types (as identified in A) in ID8-WT (blue) and STOSE (red) samples.
